# Gene Rearrangement and Modification of Immunity Factors Are Correlated with the Insertion of Bacteriocin Cassettes in Streptococcus mutans

**DOI:** 10.1128/spectrum.01806-21

**Published:** 2022-05-23

**Authors:** Mi Nguyen-Tra Le, Miki Kawada-Matsuo, Hitoshi Komatsuzawa

**Affiliations:** a Department of Bacteriology, Hiroshima University Graduate School of Biomedical and Health Sciences, Hiroshima, Japan; The National University of Singapore and the Genome Institute of Singapore

**Keywords:** bacteriocins, *Streptococcus mutans*, nisin A, mutacin, immunity factors, gene rearrangement

## Abstract

Bacteriocins have been applied in the food industries and have become promising next-generation antibiotics. Some bacteria produce bacteriocins and possess immunity factors for self-protection. Nisin A, a bacteriocin produced by Lactococcus lactis, shows broad-spectrum activity. However, the evolution and cross-resistance ability of the immunity factors in some species results in reduced susceptibility to bacteriocins. Here, we investigated the elements responsible for nisin A resistance in Streptococcus mutans and their contribution to mutacins (bacteriocins produced by S. mutans) resistance. We classified the nisin A-resistance regions into six types based on the different combinations of 3 immunity factors, *mutFEG, nsrX,* and *mutHIJ*, and the presence of mutacin synthesis operon upstream of *mutF*. Data shows that NsrX effectively acts against nisin A but not mutacins, while the newly identified ABC transporter MutHIJ acts against three mutacins but not nisin A. Three types of MutFEG are identified based on their amino acid sequences: α (in Nsr-types C and D-I), β (in Nsr-types B and d-III), and γ (in Nsr-type E). MutFEG-α strongly contributes to mutacin I resistance, while MutFEG-β and MutFEG-γ strongly contribute to mutacin III, IIIb, and nisin A resistance. Additionally, *mutFEG*-like structures could be found in various streptococcal species isolated from the oral cavity of humans, chimpanzees, monkeys, bears, and hamsters. Our findings suggest that immunity factors rearrange and adapt in the presence of bacteriocins and could be transferred among closely related species, thus altering the bacterial competition within the microflora.

**IMPORTANCE** Streptococcus mutans is an important organism of oral microbiota and associated with dental caries and systemic diseases such as stroke and endocarditis. They produce bacteriocins known as mutacins to compete with other oral bacteria and possess immune factors for self-protection. We found that the nisin A and mutacins resistance patterns correlated with the immunity components and MutFEG variants, and the genetic difference was driven by the insertion of mutacin-synthesis cassettes. Our study provides an understanding of the development of bacteriocin resistance among streptococcal species, which may alter the bacterial interaction and ecology within the oral biofilm.

## INTRODUCTION

Streptococcus mutans is among the major etiological agents of human dental caries. Various factors contributing to its pathogenicity have been reported, including adhesion, biofilm formation, water-insoluble glucan synthesis, acidogenicity, acid tolerance, and sugar metabolism at low pH (1, 2). In addition, S. mutans has been reported as a causative pathogen of infective endocarditis, where the S. mutans from severe dental caries could transfer to the heart tissue, causing heart valve injury (3, 4). Strikingly, Cnm, a collagen-binding protein, has recently been reported as an important virulence factor in systemic infections caused by S. mutans. Via bacteremia, Cnm-positive S. mutans adheres to injured blood vessels, inhibiting the repair and resulting in cerebral hemorrhage (5–7). Therefore, S. mutans is an important pathogen in both oral infectious diseases and systemic diseases. Recently, research on microflora has been more topical than ever before, and many reports regarding the relationship between microflora and diseases have been conducted (8–10).

Bacteriocins are antimicrobial peptides produced by bacteria, especially Gram-positive bacteria such as Lactococcus, Lactobacillus, Streptococcus, and Enterococcus species (11, 12). Bacteriocins are important factors allowing these oral bacteria to outcompete others in the acquisition and/or maintenance of particular niches. Bacteriocins have not only been used in food preservation and food industries but also served as a promising therapeutic for infections and diseases caused by antibiotic-resistant bacteria (13). Nisin is a well-known bacteriocin produced by Lactococcus lactis (14) and has commercially been produced for food preservation with the FDA approval as a safe product (13). Nisin A binds to lipid II, which is involved in cell wall biosynthesis and ultimately causes the formation of pores or disturbances in bacterial membranes (15, 16). In S. mutans, several bacteriocins know as mutacins, including mutacins I, II, III, B-Ny266, IV, Smb, and K8, have been identified (17, 18). Previously, we demonstrated the diversity of bacteriocins among 125 S. mutans strains (18). Each bacteriocin showed different antibacterial activity against several oral streptococcal species and other oral bacteria such as Staphylococcus aureus, Peptostreptococcus anaerobius, Parvimonas micra, Bifidobacterium dentium, Cutibacterium acnes, Actinomyces viscosus, Corynebacterium matruchotii, Campylobacter rectus, and Aggregatibacter actinomycetemcomitans. Mutacins I, II, III, and IIIb (also known as B-Ny266) showed stronger antibacterial activity against S. mutans, *P. anaerobius*, *B*. dentium, *A. viscosus*, *C. rectus*, and S. aureus than the other mutacins, including Mutacin IV, mutacin K8, and mutacin Smb. Therefore, it is speculated that bacteriocins affect the bacterial composition of the oral flora (18). In addition, several two-component systems (TCSs) (bacterial-specific signal transduction systems) have been reported to be associated with resistance to antibacterial agents, including bacteriocins (19–26). A TCS comprises two factors: a histidine kinase, for sensing environmental stimuli such as pH, cell density, and antibacterial agents, and a response regulator regulating the expression of several genes for adaptation to stimuli (27). In S. mutans, 14 sets of TCSs have been identified (28). We previously reported two TCSs involved in the resistance to nisin A produced by L. lactis and nukacin ISK-1 produced by Staphylococcus warneri (26). NsrRS is responsible for nisin A resistance by regulating the expression of *nsrX*, which is located upstream of *nsrRS* and shows binding affinity for nisin A. Additionally, we found that the expression of NsrX and MutFEG, an ABC transporter encoded by an operon located upstream of *nsrX*, is induced by nisin A. However, since the strain UA159 carried a truncated *mutE*, which resulted in an incomplete function of MutFEG transporter, we could not identify any relationship between the MutFEG transporter and resistance to nisin A in that study.

In this study, we investigated the variations in the nisin resistance (Nsr) region, defined as the gene cluster from *mutF* to *nsrS*, among 124 S. mutans strains. Interestingly, the insertion of mutacin I, III, or IIIb biosynthesis genes into the region between *alaS* (encoded an alanyl-tRNA synthetase protein) and the Nsr region resulted in the rearrangement and modification of transporter genes, leading to alterations in bacteriocin susceptibility. Furthermore, in mutacin I-producing strains, we found that *orfXYZ* contributed to resistance against mutacins I, III, and IIIb. Because we newly identified the function of *orfXYZ* as an immunity factor for mutacins, we have now designated it *mutHIJ*.

## RESULTS

### MIC of nisin A among 124 S. mutans strains.

**FIG 1:**
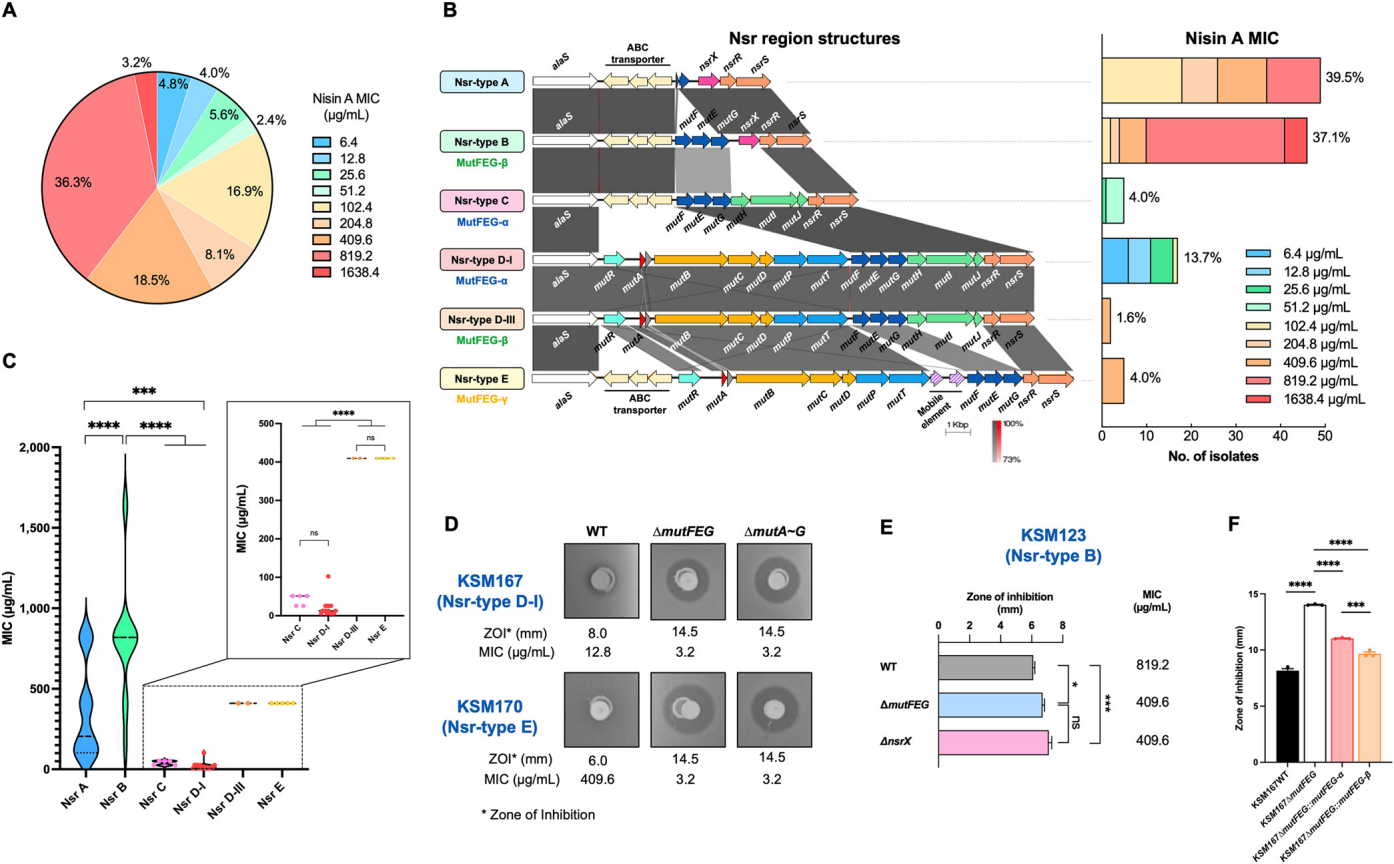
Nisin A resistance and gene cluster involved in nisin A resistance in 124 S. mutans isolates. (A) Distribution of nisin A MICs among 124 S. mutans isolates. (B) Variations in the gene cluster involved in nisin A resistance (Nsr region). The gene clusters flanked by *alaS* and *nsrS* were extracted from whole-genome sequences of 124 S. mutans strains to analyze the gene cluster composition. The gene cluster composition of Nsr-type D-I and d-III are similar; however, the amino acid sequences of MutEG between the two structures were distinct (< 32% identity). Therefore, they were separated into two groups and were designated based on the mutacin type that they carry: Nsr-type D-I (carries the mutacin I-synthesis locus) and Nsr-type d-III (carries the mutacin III-synthesis locus). One representative isolate from each Nsr type was used for the alignment to observe homology. Gray scale indicates the % sequence identity. The distribution of nisin A MIC values in each Nsr type is displayed on the right side. (C) Nisin A susceptibility among different Nsr types. Mean value and distribution of nisin A MIC of isolates from the same Nsr type was calculated. One-way ANOVA was performed to determine statistically significant differences between Nsr types. *, *P* < 0.05; **, *P* < 0.01; ***, *P* < 0.001; ****, *P* < 0.0001. (D) Role of MutFEG in nisin A resistance in Nsr-types D-I and E. MIC and soft-overlay assays were performed to investigate nisin A resistance in WT, *ΔmutFEG*, and *ΔmutA~G* bacteria. L. lactis ATCC11454 was used as a nisin A-producing strain. KSM167 and KSM170 were used as the indicator strains of Nsr-type D-I and Nsr-type C, respectively. (E) Role of MutFEG and NsrX in nisin A resistance in Nsr-type B. MIC and soft-overlay assays were performed to investigate nisin A resistance in WT, *ΔmutFEG*, and *ΔnsrX* bacteria. (F) Comparison of nisin A resistance ability between MutFEG-α and MutFEG-β. A soft-overlay assay was performed using the Δ*mutFEG* mutant of KSM167 (Nsr-type D-I) complemented with its own *mutFEG* (*mutFEG-*α) or *mutFEG-β* integrated into the chromosome under the control of a constitutive promoter. KSM167 WT and KSM167Δ*mutFEG* were used as controls. ***, *P* < 0.001; ****, *P* < 0.0001.

The MIC of nisin A varied among the 124 S. mutans strains. The MIC values ranged between 6.4 and 1638.4 μg/mL. The most prevalent MIC value was 819.2 μg/mL (36.3%) (Fig. 1A).

### The Nsr region are classified into six types.

Since we found variation in the nisin A MIC among strains, we investigated the Nsr region using previously obtained whole-genome sequences (18). The Nsr region sequences were clearly divided into 6 groups based on their gene composition and *mutFEG* sequences, designated types A, B, C, D-I, d-III and E (Fig. 1B). Nsr-type A consists of *nsrRS*, a TCS; *nsrX*, which is reported to be an immunity factor for nisin A (26); and sequences homologous to the 3′ end of *mutG* and the 5′ end of *mutF*. Nsr-type B consists of *nsrXRS* and *mutFEG*, an ABC transporter. Nsr-type C consists of *nsrRS*; *mutHIJ*, showing homology to an ABC transporter; and *mutFEG*. Nsr-types D-I and d-III carry the same structures, consisting of *nsrRS*, *mutHIJ*, and *mutFEG*; they were differed by the nucleotide sequences of *mutFEG.* Nsr-type E consists of *nsrRS* and *mutFEG*. Notably, between *alaS* and the promoter region of *mutF*, there is a set of three genes encoding an ABC transporter of unknown function (in reverse orientation) in Nsr-type A, B, and C strains, while this ABC transporter is replaced with a mutacin I- or III-synthesis cassette (*mutRAA'BCDPT*) in Nsr-type D-I and d-III strains, respectively. Interestingly, in Nsr-type E strains, both the ABC transporter in reverse orientation and the mutacin IIIb synthesis cassette coexist in this region, followed by two additional mobile elements upstream of *mutF.* The numbers of strains belonging to Nsr-types A, B, C, D-I, d-III, and E were 49 (39.5%), 46 (37.1%), 5 (4.0%), 17 (13.7%), 2 (1.6%), and 5 (4.0%), respectively (Fig. 1B).

Because the strains belonging to Nsr-types D-I, d-III, and E possessed mutacin-coding genes adjacent to the Nsr region, we next investigated the distribution of different mutacin types (mutacins I, II, III, IIIb, IV, Smb and K8) within each Nsr type (Tables S1 and S2). The numbers of strains carrying single, double, and triple mutacin genes were 84 (67.7%), 23 (18.5%), and 4 (3.2%), respectively, while 13 strains carried none of the mutacins of interest. Mutacins I, III, and IIIb were exclusively present in Nsr-type D-I, d-III, and E strains, while mutacins IV, Smb, and K8 were exclusively present in Nsr-type A, B, and C strains (Table S1). Mutacin II was distributed in both Nsr-type A and D-I strains. Since the strains with Nsr-types A and B accounted for the majority (76.6%) of this collection, we next compared the distribution of different mutacins between the strains belonging to these two Nsr types (Table 1). Mutacin K8 (single positive) and mutacin Smb (single positive) were significantly more prevalent among Nsr-type A than among Nsr-type B (*P = *0.0004 and *P = *0.031, respectively). In contrast, mutacin IV (single positive or co-exists with mutacins K8/Smb) were significantly more prevalent among Nsr-type B than among Nsr-type A (*P = *0.000665 and *P = *0.000016, respectively).

**TABLE 1:** Comparison of mutacin traits between Nsr type A and Nsr type B^*a*^

Mutacin type	Total (*n* = 95)	Nsr type a (*n* = 49)	Nsr type B (*n* = 46)	OR (95%CI)	*P* value
Mutacin II (single positive)	3 (3.2%)	3 (6.1%)	0 (0.0%)	NA	0.243
Mutacin IV	55 (57.9%)	18 (36.7%)	37 (80.4%)	0.141 (0.056 - 0.359)	0.000016****
Mutacin IV (single positive)	37 (38.9%)	11 (22.4%)	26 (56.5%)	0.223 (0.092 - 0.542)	0.000665***
Mutacin IV + Smb	14 (14.7%)	4 (8.2%)	10 (21.7%)	0.320 (0.093 - 1.105)	0.062
Mutacin IV + K8	2 (2.1%)	1 (2.0%)	1 (2.2%)	NA	1.000
Mutacin IV + Smb + K8	2 (2.1%)	2 (4.1%)	0 (0.0%)	NA	0.495
Mutacin Smb	26 (27.4%)	15 (30.6%)	11 (23.9%)	1.404 (0.565 - 3.487)	0.464
Mutacin Smb (single positive)	9 (9.5%)	8 (16.3%)	1 (2.2%)	NA	0.031*
Mutacin Smb + K8	1 (1.1%)	1 (2.0%)	0 (0.0%)	NA	1.000
Mutacin K8	20 (21.1%)	18 (36.7%)	2 (4.3%)	12.774 (2.762 - 59.080)	0.000109***
Mutacin K8 (single positive)	15 (15.8%)	14 (28.6%)	1 (2.2%)	18.000 (2.257 - 143.547)	0.000422***
No mutacin	12 (12.6%)	5 (10.2%)	7 (15.2%)	0.633 (0.186 - 2.157)	0.462

a *P* values were calculated using Chi-square test and Fisher’s exact test for differences in the prevalence of each feature (in rows). NA, test not applicable (one or more values equal to zero). OR, odds ratio; CI, confidence interval. *, *P* < 0.05; **, *P* < 0.01; ***, *P* < 0.001; ****, *P* < 0.0001.

Next, we investigated the relationship between Nsr type and nisin A susceptibility. The results indicated that the susceptibility to nisin A varied among Nsr types (Fig. 1C). Nsr-type B exhibited the highest MIC among the 6 Nsr-types. Nsr-types A, d-III and E showed higher MICs than Nsr-types C and D-I (*P < *0.0001) and lower MICs than Nsr-type B (*P < *0.0001).

### MutFEG and/or NsrX are responsible for nisin A resistance.

We previously reported that NsrX was responsible for nisin A resistance (26). However, the strains of Nsr-types d-III and E did not possess NsrX but showed a similar MIC to Nsr-type A, which possessed NsrX. Therefore, we tried to find out which factors are involved in nisin A resistance in the strains without *nsrX*. We constructed knockout mutants of *mutFEG* in KSM167 (Nsr-type D-I) and KSM 170 (Nsr-type E). Additionally, several KSM167 mutants with deletions of various genes, including *mutHIJ*, were constructed. The nisin A susceptibility of Δ*mutFEG* mutants in KSM167 and KSM170 was increased relative to that of each WT strain (Fig. 1D). On the other hand, the Δ*mutHIJ* mutant of KSM167 showed the same nisin A susceptibility as the WT (Fig. S1). Other mutants, such as KSM167*ΔmutP-G*, *ΔmutP-J*, *ΔmutA-G*, and *ΔmutA-J*, in which *mutFEG* was included in the deleted regions, also showed an increase in nisin A susceptibility to a level equal to that of Δ*mutFEG* (Fig. S1), suggesting that the increased susceptibility to nisin A was due to the loss of *mutFEG*.

In addition, we constructed a Δ*mutFEG* mutant and an Δ*nsrX* mutant in an Nsr-type B strain. Both mutants exhibited increased susceptibility to nisin A (Fig. 1E). The nisin A MIC values of Δ*mutFEG* and Δ*nsrX* mutants were decreased to an equal extent (from 819.2 μg/mL in the WT to 409.6 μg/mL in each mutant, Fig. 1E) but were still considerably higher than those of the Nsr-types D-I and E Δ*mutFEG* mutants (3.2 μg/mL, Fig. 1D).

### MutFEG are classified into three types based on their amino acid sequences.

The amino acid sequences of MutF, MutE, and MutG differed to some extent among the different Nsr types (Table 2 and Fig. S2). The MutF sequences from Nsr-types B, C, D-I and d-III were close to identical (≥99.14%), with the exception of one amino acid (isoleucine) addition at the 2nd position in Nsr-type C and one amino acid difference at the 30^th^ amino acid in Nsr-types B and d-III (arginine) and Nsr-types C and D-I (histidine) (Fig. S2A). MutF from Nsr-type E showed approximately 94% identity to the sequences from the other types, with a 13-amino acid difference. On the other hand, the MutE and MutG sequences from Nsr-types C and D-I exhibited 100% and 99.59% identity, respectively (Table 2). Similarly, the MutE and MutG sequences from Nsr-types B and d-III showed 99.60% and 99.19% identity, respectively, but were markedly less similar to those from Nsr-types C and D-I (only 16.40% and 31.57% identity, respectively, Table 2). MutE and MutG from Nsr-type E were distinct from those of the other types but were more similar to those from Nsr-types B and d-III (≥86.40% and ≥83.13%, respectively) than to those from Nsr-types C and D-I (18.80% and 31.17%, respectively, Table 2). Based on the MutFEG similarity among Nsr types, we further classified the MutFEG sequences into 3 types, designated MutFEG-α (including Nsr-types C and D-I), MutFEG-β (including Nsr-types B and d-III), and MutFEG-γ (including only Nsr-type E).

**TABLE 2:** Percent amino acid sequences identity of MutFEGXY among different Nsr types^*a*^

MutFEG	Size (aa)	% Identity in:				
		Nsr-type B	Nsr-type C	Nsr-type D-I	Nsr-type D-III	Nsr-type E
MutF						
Nsr-type B	233					
Nsr-type C	234	99.14%				
Nsr-type D-I	233	99.57%	99.57%			
Nsr-type D-III	233	100.00%	99.14%	99.57%		
Nsr-type E	233	94.01%	93.58%	94.01%	94.01%	
MutE						
Nsr-type B	249					
Nsr-type C	251	16.40%				
Nsr-type D-I	251	16.40%	100.00%			
Nsr-type D-III	249	99.60%	16.40%	16.40%		
Nsr-type E	249	86.40%	18.80%	18.80%	86.80%	
MutG						
Nsr-type B	248					
Nsr-type C	246	31.57%				
Nsr-type D-I	246	31.57%	99.59%			
Nsr-type D-III	248	99.19%	31.57%	31.57%		
Nsr-type E	248	83.53%	31.17%	31.17%	83.13%	
MutH						
Nsr-type D-III	246			100%		
MutI						
Nsr-type D-III	640			99.21%		
MutJ						
Nsr-type D-III	118			98.31%		

a The % identity indicates the number of matching amino acids/total number of amino acids.

### Different nisin A resistance abilities associated with different types of *mutFEG* sequences.

Since the MutFEG amino acid sequences were mainly classified into 3 groups, we tried to construct the complementation strains with different types of *mutFEG.* The three types of *mutFEG* were individually cloned under the control of the *P_ftf_* constitutive promoter (26), however, we could not obtained the *mutFEG-γ* cassette due to unknown reason. The obtained cassettes of *mutFEG-α* and -*β* were reinserted into the chromosomes of KSM167Δ*mutFEG-α* mutants to create KSM167*ΔmutFEG-α::mutFEG-α* and KSM167*ΔmutFEG-α::mutFEG-β*. The results showed that the strains complemented with the β-type of *mutFEG* exhibited a significantly smaller zone of inhibition in response to nisin A than the strain carrying α-type *mutFEG* (*P = *0.0002, Fig. 1F), suggesting that *mutFEG-β* confers a higher resistance ability against nisin A than *mutFEG-α*. The difference in nisin A resistance conferred by the *mutFEG-α* and *mutFEG-β* cassettes to the complemented mutant is consistent with the resistance shown by the respective WT strains harboring these elements (Fig. 1B and C).

### Different mutacins I, III, and IIIb susceptibility patterns among Nsr types.

**FIG 2:**
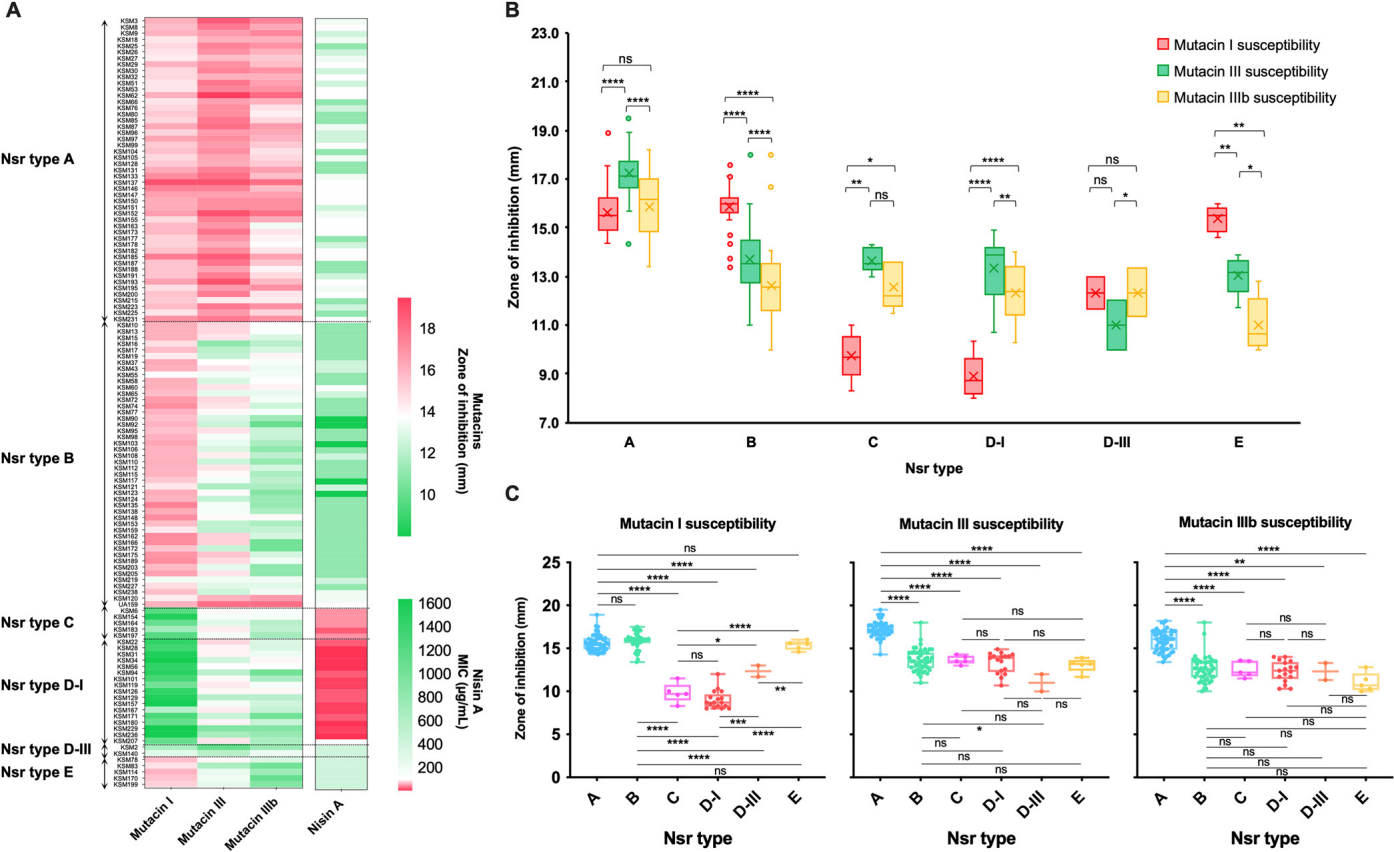
Mutacin I, III, and IIIb susceptibility in different Nsr types. (A) The mutacin I, III, and IIIb and nisin A susceptibility of 124 S. mutans isolates. The mutacin and nisin A susceptibility pattern of each isolate are shown according to Nsr type. A soft-agar overlay assay was performed to evaluate the susceptibility to mutacins I, III, and IIIb using KSM167, KSM2, and KSM170, respectively, as the bacteriocin-producing strains. All 124 S. mutans strains were used as indicator strains (*n* = 3). (B, C) Mean resistance of each Nsr type against mutacins I, III, and IIIb. The distribution of the zone of inhibition (mm) of each Nsr type is shown, and One-way ANOVA was performed to determine statistically significant differences in susceptibility to different mutacins within the same Nsr type (B) or susceptibility to the same mutacin among different Nsr types (C), respectively. *, *P* < 0.05; **, *P* < 0.01; ***, *P* < 0.001; ****, *P* < 0.0001.

The differences in nisin A susceptibility among the different Nsr types prompted us to investigate their susceptibility to mutacins I, III, and IIIb via the soft agar overlay assay using all 124 isolates as overlay target strains. The mutacin I, III, and IIIb and nisin A susceptibility patterns of each isolate of different Nsr types are shown in Fig. 2A, and the mean zone of inhibition of each type are shown in Fig. S3. Among three mutacins, Nsr-types C and D-I exhibited resistance against mutacin I but significantly higher susceptibility to mutacins III and IIIb (*P < *0.05 in Nsr-type C and *P < *0.0001 in Nsr-type D-I, Fig. 2B). In contrast, Nsr-types B and E showed significantly higher susceptibility to mutacin I than to mutacin III and IIIb (*P < *0.0001 in Nsr-type B and *P < *0.01 in Nsr-type E). The ratios of the zone of inhibition of mutacin I/mutacin III and mutacin I/mutacin IIIb in Nsr-types C and D-I ranged between 0.67–0.77, while ranging between 1.15–1.39 in Nsr-types B and E (Fig. S3).

Among 6 Nsr types, Nsr-types C and D-I exhibited lower susceptibility to mutacin I than any other Nsr type (*P < *0.001, Fig. 2C). Nsr-types d-III and E exhibited the lowest susceptibility to mutacin III and mutacin IIIb, respectively, although there were no statistical differences when compared to Nsr-types C and D-I (Fig. 2C), maybe due to the low number of Nsr types d-III and E isolates (*n* = 2 and *n* = 5, respectively). The ratios of the zone of inhibition of mutacin III/mutacin IIIb in Nsr types d-III and E were in the reverse direction (0.89 and 1.18, respectively), while this ratio was relatively constant among Nsr types A, B, C, and D-I, which was around 1.09 (Fig. S3). These data infer that mutacin III generally has a little higher activity than mutacin IIIb (in Nsr types A, B, C, and d-III), but Nsr-type d-III has specific resistance against mutacin III, and Nsr-type E has specific resistance against mutacin IIIb.

Nsr-type A displayed high susceptibility to all three mutacin types, especially to mutacins III and IIIb, which were significantly higher than those of any other Nsr-type (*P < *0.01, Fig. 2C).

### MutFEG and/or MutHIJ are responsible for mutacins I, III, and IIIb resistance.

**FIG 3:**
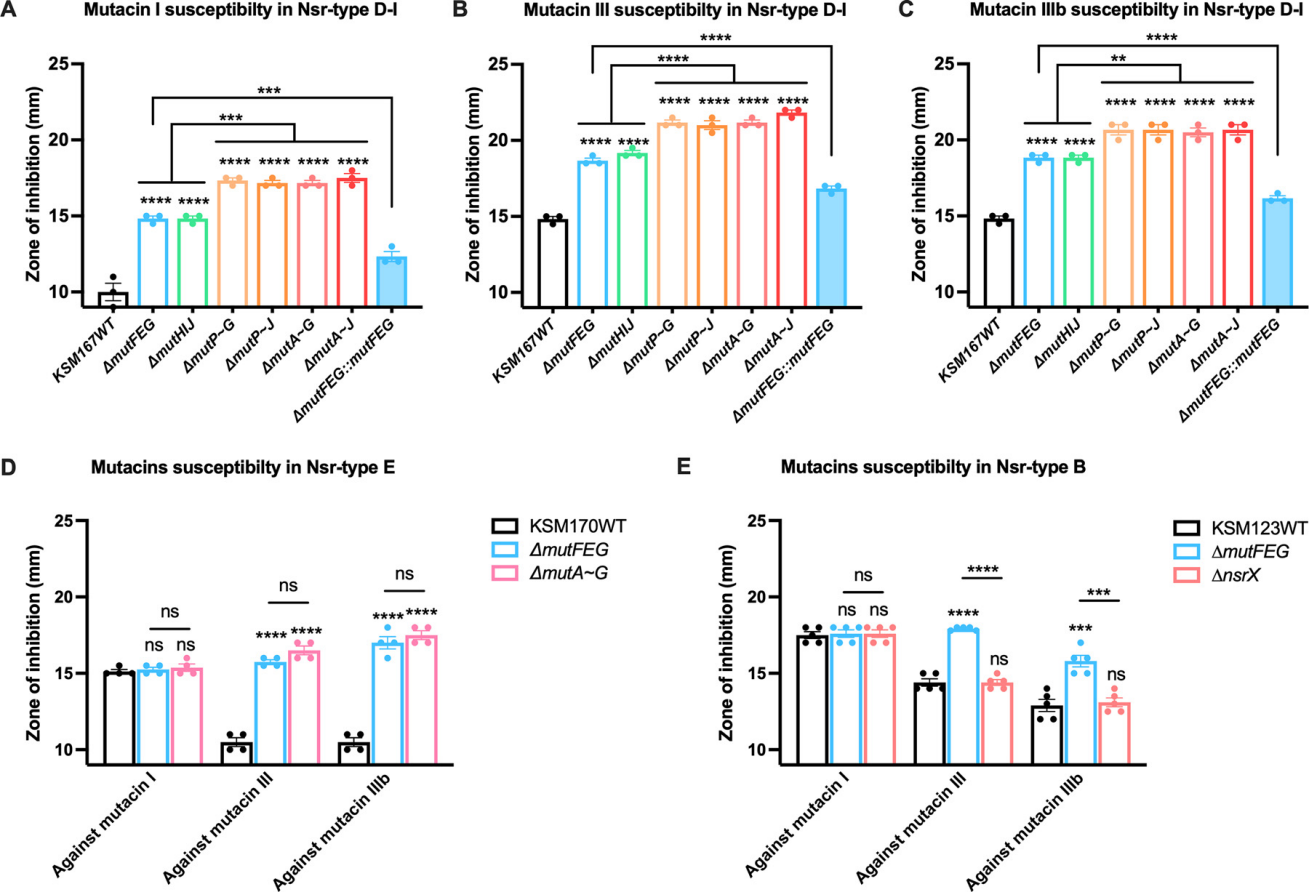
Susceptibility to mutacins I/III/IIIb in WT bacteria and various deletion mutants. Soft agar overlay assays were performed to evaluate the susceptibility to mutacins I, III, and IIIb in various knockout mutants from different Nsr types. Data are presented as mean ± SEM. One-way ANOVA was performed to determine statistically significant differences between strains. *, *P* < 0.05; **, *P* < 0.01; ***, *P* < 0.001; ****, *P* < 0.0001. (A–C) Immunity factors involved in resistance to mutacins I, III, and IIIb in the Nsr-type D-I isolate (KSM167). Several KO mutants and complemented strains derived from KSM167 (Nsr-type D-I) were constructed, including Δ*mutFEG*, Δ*mutHIJ*, Δ*mutP~G*, Δ*mutP~J*, Δ*mutA~G*, Δ*mutA~J*, and Δ*mutFEG::mutFEG*. (D) Immunity factors involved in resistance to mutacins I, III, and IIIb in the Nsr-type E isolate (KSM170). Δ*mutFEG* and Δ*mutA~G* mutants derived from KSM170 (Nsr-type E) were constructed. (E) Immunity factors involved in resistance to mutacins I, III, and IIIb in the Nsr-type B isolate (KSM123). Δ*mutFEG* and Δ*nsrX* mutants derived from KSM123 (Nsr-type B) were constructed.

To identify the factors associated with mutacin I, III or IIIb resistance, we investigated the susceptibility of several KO mutants derived from KSM167 (Nsr-type D-I) to mutacins. Compared to the WT, the Δ*mutFEG* and Δ*mutHIJ* mutants showed similarly increased susceptibility to mutacins I, III, and IIIb, and Δ*mutA-J* (which included the deletion of both *mutFEG* and *mutHIJ*) showed further increased susceptibility to mutacins (Fig. 3A to C). Additionally, the Δ*mutA-G* and Δ*mutP-G* mutants showed the same susceptibility as the Δ*mutA-J* and Δ*mutP-J* mutants. In the KSM167 WT, the susceptibility to mutacins III and IIIb was higher than that to mutacin I. These data suggested that MutFEG and MutHIJ are responsible for mutacin I, III, and IIIb resistance, while the gene cluster from *mutA* to *mutT* may not involve in the resistance.

To verify whether *mutFEG* and *mutHIJ* are cotranscribed, we performed PCR to amplify the junctions between *mutG* and *mutH* and between *mutT* and *mutF* using cDNA of KSM167. The predicted size of PCR fragment was generated from the amplification of *mutG* to *mutH*, while no PCR fragment could be obtained from the amplification of *mutT* to *mutF* (Fig. S4A), suggesting that *mutFEG* and *mutHIJ* were cotranscribed in a single operon. Next, the expression of *mutH* in the Δ*mutFEG* and Δ*mutA-G* mutants was investigated. Since the Δ*mutFEG* mutant was constructed via the replacement of *mutFEG* with the erythromycin resistance gene, the mutant still possessed the promoter region upstream of *mutFEG*. The expression of *mutH* in the Δ*mutFEG* mutant was increased 20-fold by nisin A addition, while its expression was constant in the mutant with *mutA-G* deletion (*P < *0.05, Fig. S4B), suggesting that *mutH* expression was regulated by the promoter upstream of *mutF*.

For the Nsr-type E, the Δ*mutFEG* and Δ*mutA-G* mutants in KSM170 significantly increased susceptibility to mutacins III and IIIb relative to the WT but did not increase susceptibility to mutacin I (Fig. 3D). These results suggest that the MutFEG-γ in Nsr-type E contributes to the resistance against mutacin III and IIIb but lacks specificity to mutacin I.

Similarly, for the Nsr-type B, the Δ*mutFEG* mutant in KSM123 also significantly increased susceptibility to mutacins III and IIIb but did not increase susceptibility to mutacin I (Fig. 3E). In contrast, the Δ*nsrX* did not alter the susceptibility to all mutacins (Fig. 3E), inferring that NsrX is not involved in mutacin resistance. MutFEG-β in Nsr-type B contributes to the resistance against mutacin III and IIIb but lacks specificity to mutacin I.

### Different mutacin I, III, and IIIb resistance abilities associated with the different types of *mutFEG*.

The complemented strains with the two types of *mutFEG* generated in the Δ*mutFEG* mutant of KSM167 were used for this evaluation. The strains complemented with *mutFEG-α* showed significantly lower susceptibility to mutacin I relative to those complemented with *mutFEG-β* (*P < *0.0001). In contrast, the strains complemented with *mutFEG-β* showed significantly lower susceptibility to mutacins III and IIIb relative to those complemented with *mutFEG-α* (*P < *0.0001 and *P < *0.001, respectively, Fig. S5).

### Role of NsrRS in nisin A and mutacins resistance.

**FIG 4:**
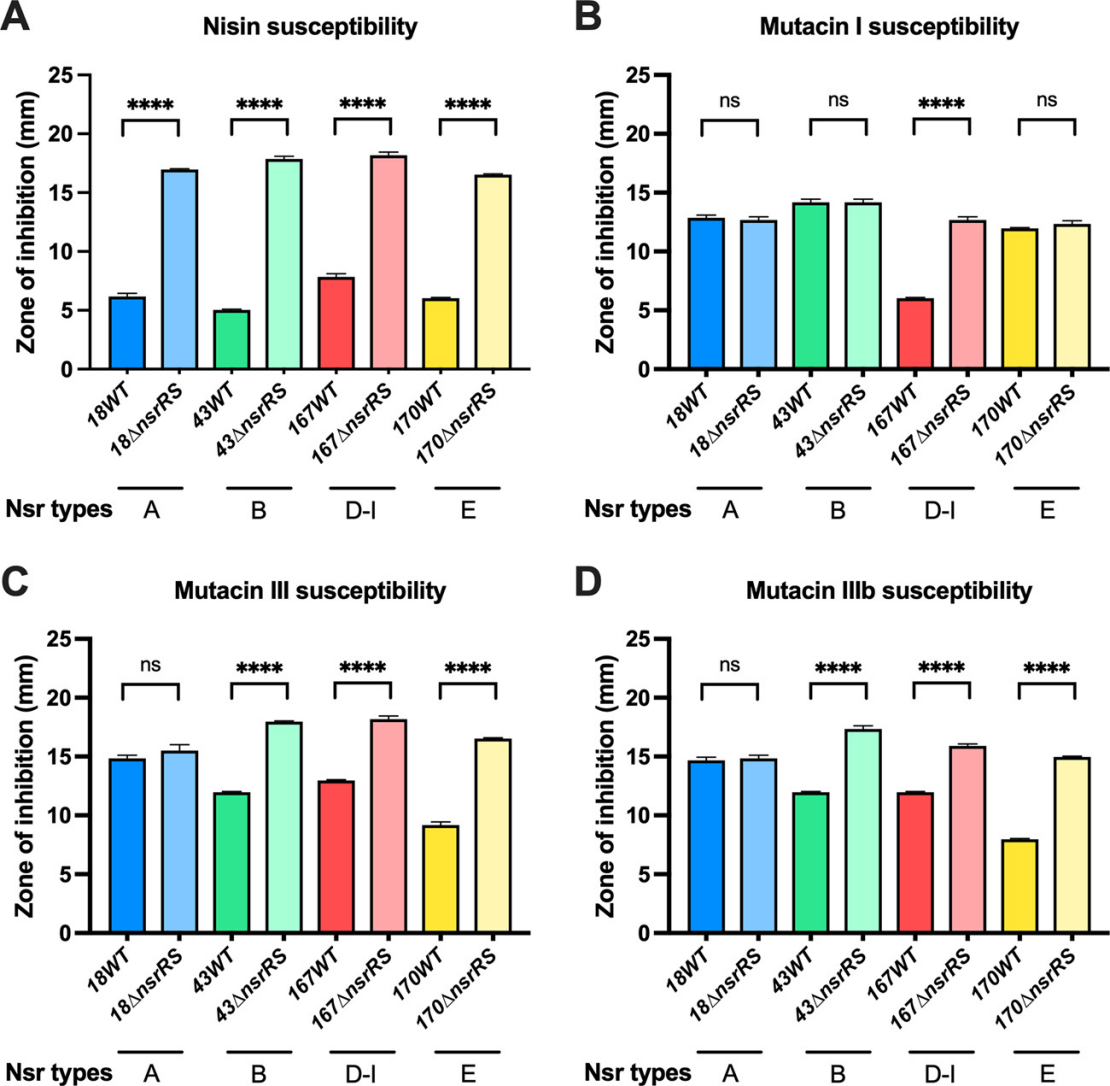
Susceptibility to nisin A and mutacins I, III, and IIIb in WT and Δ*nsrRS* mutants. Δ*nsrRS* mutants of Nsr-types A, B, D-I, and E were constructed. A soft agar overlay assay was performed to evaluate the susceptibility to nisin A (A) and mutacins I (B), III (C), and IIIb (D) in the WT and mutant strains. Data are represented as mean ± SEM (*n* = 3). One-way ANOVA was performed to determine statistically significant differences between strains. *, *P* < 0.05; **, *P* < 0.01; ***, *P* < 0.001; ****, *P* < 0.0001.

Since the NsrRS TCS is required for activating the *nsrX* expression in response to nisin A in UA159 (Nsr-type B) (26), we tried to determine the role of this TCS in response to nisin A and mutacins in all Nsr types. We attempted to construct Δ*nsrRS* mutants of all 6 Nsr types; however, no Δ*nsrRS* mutant of Nsr-type C or Nsr-type d-III could be obtained. Δ*nsrRS* mutants of the 4 other Nsr types were successfully generated. The results demonstrated that the Δ*nsrRS* mutants significantly increased nisin A susceptibility in all 4 Nsr types (A, B, D-I, and E) relative to the WT (*P < *0.0001, Fig. 4A). On the other hand, regarding mutacin I susceptibility, the Δ*nsrRS* mutants only increased mutacin I susceptibility in Nsr-type D-I (*P < *0.0001) and not in Nsr-type A, B, or E (Fig. 4B). The Δ*nsrRS* mutants of Nsr-types B, D-I, and E displayed significantly higher susceptibility to mutacin III or IIIb than the WT (*P < *0.0001), while Nsr-type A did not (Fig. 4C and D).

### Low expression of *mutF* and *nsrX* in Δ*nsrRS* mutants upon the addition of nisin A.

To determine the association of NsrRS with the expression of immunity factors induced by nisin A, we investigated their expression in the Δ*nsrRS* mutants of each Nsr type. The WT strains of Nsr-types A, B, D-I, and E showed significantly increased expression of *mutFEG* and/or *nsrX* upon the addition of nisin A compared to the Δ*nsrRS* mutants (Fig. S6A, B). In addition, the *mutA* genes of the Nsr-type D-I and E strains were not expressed even in the presence of nisin A (Fig. S6B).

### Phylogenetic analysis.

**FIG 5:**
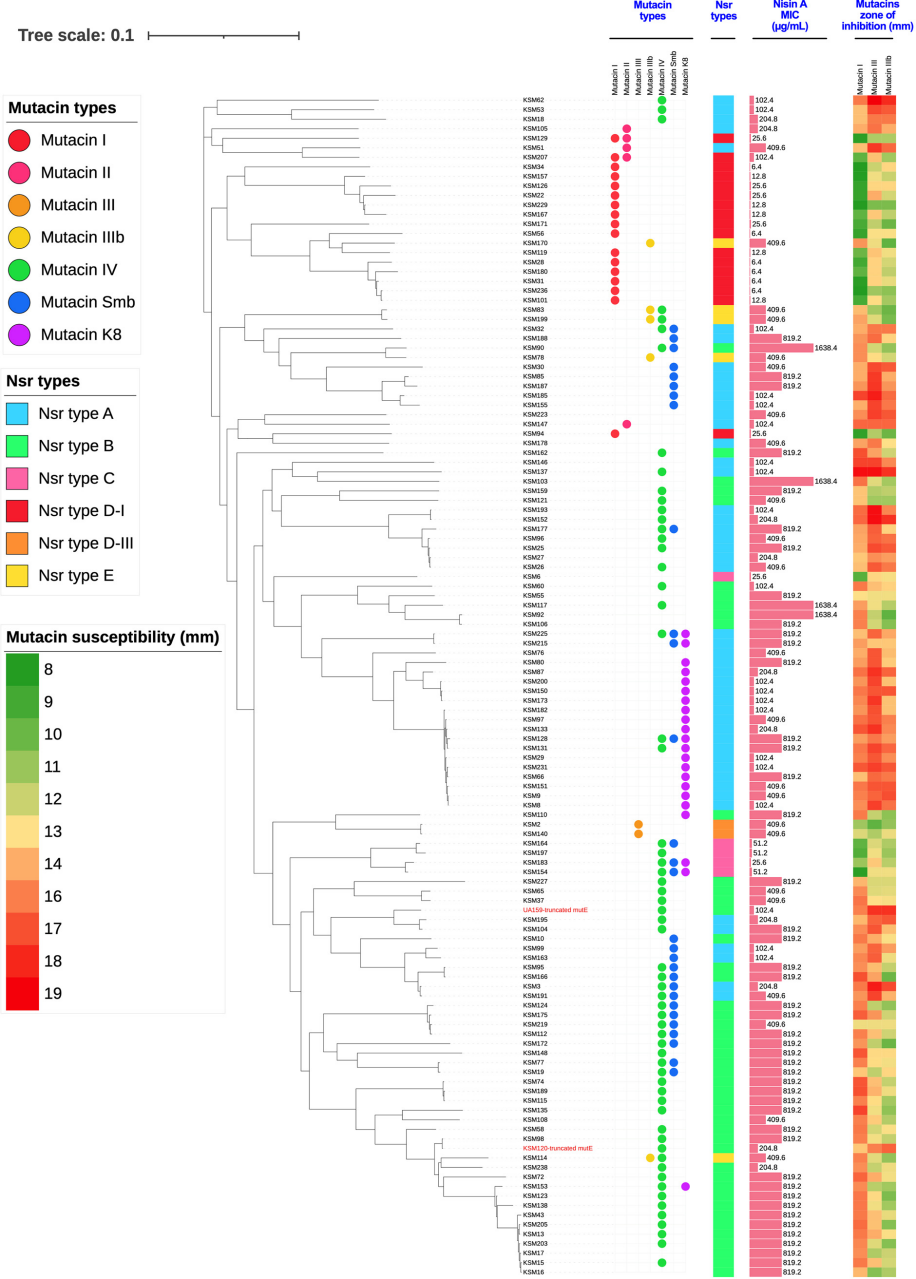
Phylogenetic analysis of S. mutans. A phylogenetic tree was constructed using 124 genome sequences. Isolates are annotated with data on mutacin types, Nsr types, nisin A MICs (μg/mL), and mutacin zones of inhibition (mm), from left to right.

A SNP-based phylogenetic tree of the whole-genome sequences of 124 isolates was generated and annotated with the data on mutacins, Nsr types, nisin A MICs, and mutacin susceptibility (Fig. 5). Most mutacin I-positive strains were classified in the same cluster, belonged to Nsr-type D-I, and showed high susceptibility to nisin A, extremely low susceptibility to mutacin I, and moderate susceptibility to mutacins III and IIIb. Two mutacin III-positive strains distributed close together in the same cluster, belonged to Nsr-type d-III, and exhibited low susceptibility to nisin and mutacin III and moderate susceptibility to mutacins I and IIIb. Five mutacin IIIb-positive strains distributed in 3 different clusters belonged to Nsr-type E and showed low susceptibility to nisin A and mutacins III and IIIb but high susceptibility to mutacin I. Interestingly, two isolates (UA159 and KSM120) belonging to Nsr-type B carried a truncated *mutE* and therefore displayed nisin A and mutacin susceptibility patterns similar to those of the Nsr-type A isolates.

### Genetic comparison among different streptococcal species.

**FIG 6:**
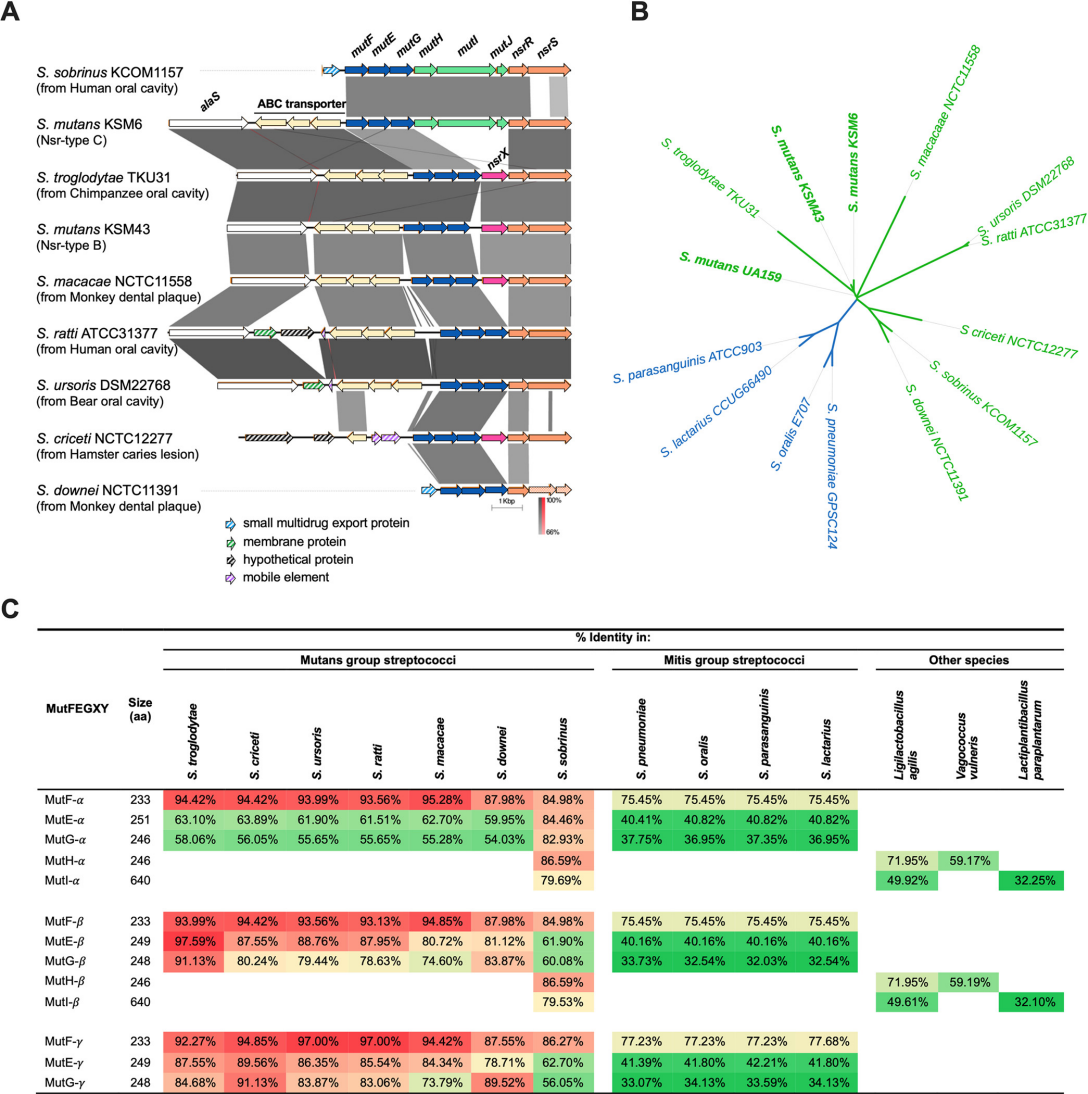
Genetic comparison among different streptococcal species. (A) Comparison of the *mutFEG-*coding region in different streptococcal species. The gene clusters surrounding *mutFEG* in *S. sobrinus* KCOM1157 (NPOU01000001), *S. troglodytae* TKU31 (NZ_AP014612.1), *S. macacae* NCTC11558 (UHFV01000002), S. ratti ATCC31377 (CP043405), *S. ursoris* DSM22768 (NZ_JABASA010000001.1), *S. criceti* NCTC12277 (NZ_UHFB01000008.1), and *S. downei* NCTC11391 (NZ_UHFA01000003.1) were extracted and compared to those of S. mutans KSM6 (Nsr-type C) and KSM43 (Nsr-type B). Grayscale indicates the % sequence identity. (B) Phylogenetic relationship among S. mutans and the other mutans group streptococci and mitis group streptococci. A phylogenetic tree was constructed using whole-genome sequences of S. mutans KSM6 and KSM43 and the other strains from the NCBI database. Green, mutans group; blue, mitis group. (C) Percent amino acid sequences identity of MutF, MutE, MutG, MutH, and MutI among different streptococcal species and some other species. The % identity indicates the number of matching amino acids/total number of amino acids.

We found that the regions encoding *mutFEG* and *nsrRS* were also present in the other mutans group streptococci such as *S. sobrinus*, *S. troglodytae*, *S. macacae*, S. ratti, *S. ursoris*, *S. criceti*, and *S. downei* (Fig. 6A). Interestingly, the gene composition of Nsr-type B was identical to that of *S. troglodytae* TKU31, a bacterium isolated from chimpanzee oral cavity (29), and *S. macacae* NCTC11558, a strain isolated from the dental plaque of monkeys (30). *S. criceti* NCTC12277, a species from hamster caries lesion, also carried *mutFEG*, *nsrX*, and *nsrRS*, but upstream of *mutFEG* were two mobile elements. S. ratti ATCC31377 and *S. ursoris* DSM22768, which were isolated from human and bear oral cavity, respectively, carried highly similar sequences, and an insertion of a membrane protein into the region between *alaS* and the ABC transporter, which could not be seen in the remaining species. Strikingly, *S. sobrinus* KCOM1157 was the only species that carries both *mutFEG* and *mutHIJ*, similar to S. mutans Nsr-type C. Meanwhile, the phylogenetic tree based on SNP analysis of whole-genome sequences (Fig. 6B) showed that S. mutans and *S. sobrinus* were more distant than the relationship between S. mutans and *S. troglodytae* or *S. macacae*.

From BLAST searching with NCBI protein database, we found the amino acid sequences of MutF-α, -β and -γ were highly similar to those from mutans group streptococci (≥84% identity, Fig. 6C), and mitis group streptococci such as S. pneumoniae, S. oralis, *S. parasanguinis*, and *S. lactarius* (≥75% identity). MutEG-β were closest to *S. troglodytae* (>91% identity), and MutEG-γ were closest to *S. criceti* (>91% identity). Meanwhile, MutEG-α exhibited high similarity only to those in *S. sobrinus* (84.46% and 82.93% identity, respectively), critically higher than to the other species (<65% identity). Additionally, MutHI was not generally present in most of the streptococcal species except for *S. sobrinus* (≥79.69% identity).

## DISCUSSION

**FIG 7:**
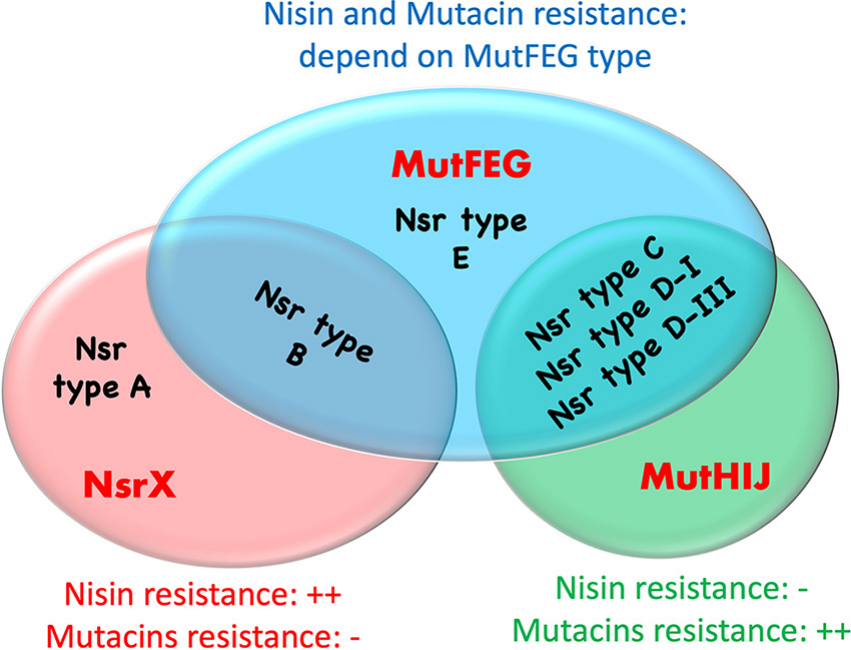
Functions of different immunity factors. Six Nsr types are generated from different combinations of three immunity factors (NsrX, MutFEG, and MutHIJ). Each kind of immunity factor shows different resistance abilities against nisin A and mutacins.

Our study demonstrated that NsrX, MutFEG, and MutHIJ were three different immunity factors, and various combinations of these factors generate different Nsr types (Fig. 7). NsrX functions against nisin A but not against mutacins, while MutHIJ functions against mutacins but not against nisin A. On the other hand, MutFEG has a partial overlapping function with Nsr X and MutHIJ since it can contribute to resistance against both nisin A and mutacins, but the extent of this resistance is dependent on the MutFEG type.

We found the clear relationships between nisin A resistance and two factors, NsrX and MutFEG. Different MutFEG types (α, β, and γ) also displayed different contributions to nisin A resistance. Nsr-type B strains showed the highest resistance to nisin A among all Nsr types due to the presence of both NsrX and MutFEG-β. The results of single-knockout experiments indicated that MutFEG and NsrX contributed equally to nisin A resistance (Fig. 1E). The lack of one factor resulted in a reduction in nisin A resistance, which was consistent with the fact that Nsr-type A showed intermediate nisin A resistance due to the presence of only NsrX (*mutFEG* was deleted), and Nsr-type d-III (MutFEG-β) and Nsr-type E (MutFEG-γ) also showed intermediate nisin A resistance due to the presence of only MutFEG (*nsrX* was deleted). In contrast, Nsr-types C and D-I displayed the lowest nisin A resistance due to the absence of NsrX and a distinct type of MutFEG (MutFEG-α).

We found that the MutFEG sequences differ among strains carrying different types of mutacin synthesis/immunity locus. Previous reports indicated that MutFEG was an immunity factor for the respective mutacins (17), but variations in MutFEG have not been investigated. MutFEG-α (in Nsr-types C and D-I) was involved in mutacin I resistance, with mutacin I susceptibility was equal to 0.6–0.7-fold of mutacin III or IIIb susceptibilities. In contrast, MutFEG-β (in Nsr-types B and d-III) and MutFEG-γ (in Nsr-type E) were more associated with mutacin III and IIIb resistance, where mutacin I susceptibility was equal to 1.12–1.17-fold of mutacin III susceptibility and to 1.00–1.39-fold of mutacin IIIb susceptibility (Fig. 2, S3, and S5). The complementation obtained in our study did not fully restore the level of resistance as the WT, maybe due to the lower expression of *P_ftf_* compared to the extremely high expression of *mutFEG* when regulated by the TCS NsrRS under the stimulation of nisin A or mutacins (*mutF* expression in KSM167WT increased over 100-fold when sensed with nisin A, Fig. S6B). However, the native promoter of each *mutFEG* type can cause unequal expression levels of these immunity factors, which may contribute to the different resistance abilities against the same mutacin. Therefore, we constructed the complementation using the same promoter *P_ftf_* to eliminate the effect of native promoters. From our data, the *mutFEG-α* and *mutFEG*-*β-* complemented mutants under *P_ftf_* displayed the inverse patterns in response to mutacin I and mutacins III/IIIb (Fig. S5), inferring that the different susceptibility derived from their different specificity against these mutacins. Besides, MutHIJ was involved in mutacin I, III and IIIb resistance but was not involved in nisin A resistance (Fig. 3A to C and S1). Although the amino acid sequence of MutIJ differed slightly between Nsr-types D-I and d-III (99.21% and 98.31% identity, respectively, Table 2), the Δ*mutHIJ* mutant of KSM167 (Nsr-type D-I) displayed equal increases in susceptibility to mutacins I, III, and IIIb (Fig. 3A to C). Therefore, MutHIJ contributed equally to mutacin I, III, and IIIb resistance.

**FIG 8:**
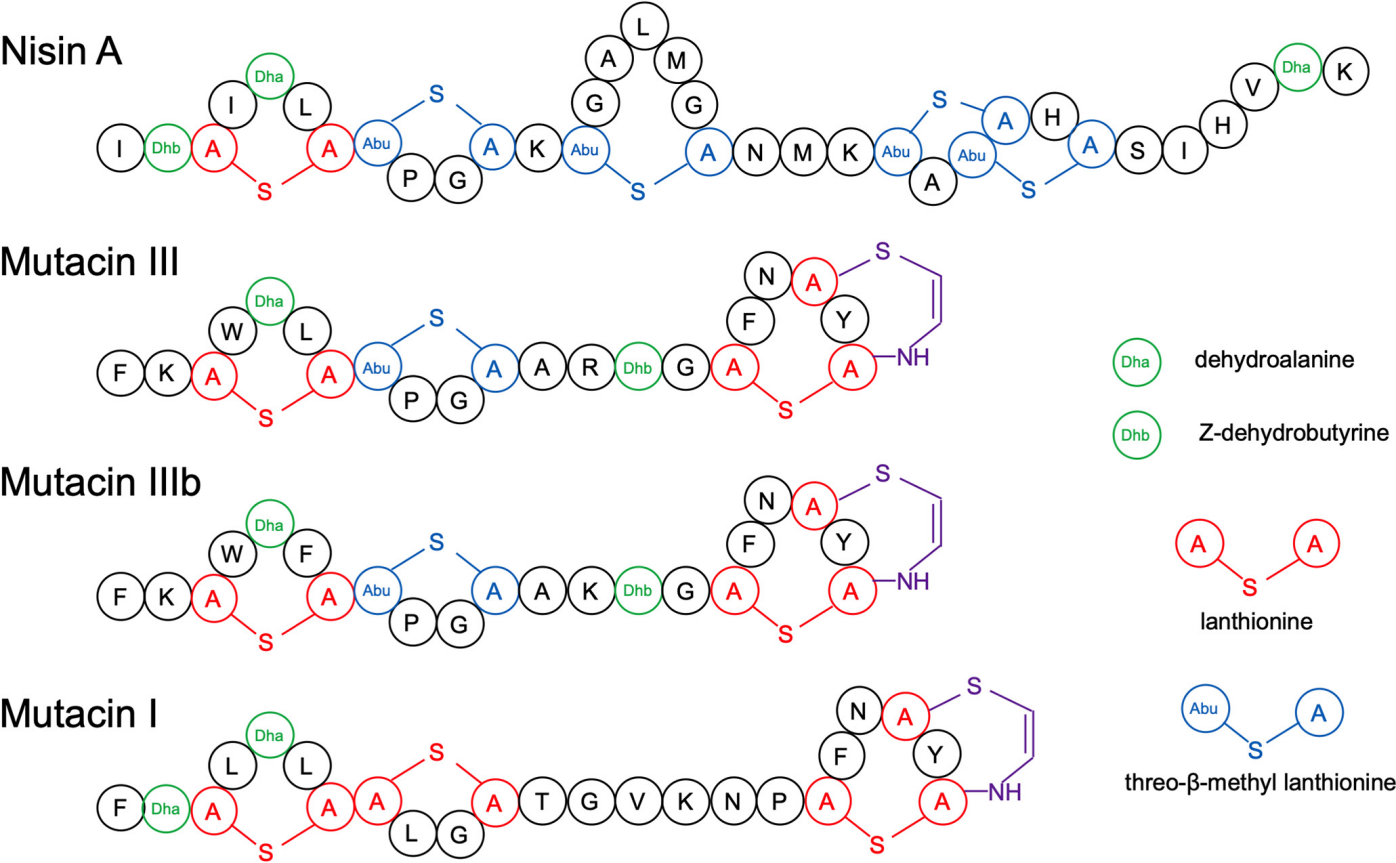
Structures of Nisin A and mutacins III, IIIb, and I.

Based on our results, we hypothesized that the structure of MutFEG was altered to adapt to different types of mutacins (I, III, and IIIb) via the introduction of several mutations. A comparison of the structures of mature mutacins I, III, and IIIb and nisin A (31–33) is provided in Fig. 8. Mutacins III and IIIb possessed almost identical structures, with the exception of differences in 2 amino acids at the 6th and 13th of the mature peptide. Although the amino acid sequences of MutFEG from Nsr-types d-III (mutacin III) and E (mutacin IIIb) shared 83.13% to 94.01% identity (Table 2), the susceptibility patterns toward each mutacin and nisin A were similar in Nsr-types d-III and E, indicating that the two MutFEG types (β and γ) were equally responsible for resistance against nisin A and mutacins III and IIIb. However, MutFEG-β and MutFEG-γ were almost incapable of resisting mutacin I owing to their low similarity to MutEG-α (≤ 31.57%, Table 2). In contrast, MutFEG-α in Nsr-types C and D-I was responsible for resistance to mutacin I better than against nisin A, mutacin III, and mutacin IIIb (Fig. 1F and 3A–C). The analysis of peptide structures further showed that the structures of the 1st and 2nd rings of mutacins III and IIIb and nisin A were nearly homologous, but the 2nd ring of mutacin I was different from those of the other peptides, suggesting that the structures of the first two rings are important for recognition by the MutFEG transporter. Interestingly, MutHIJ was involved in resistance to mutacins I, III, and IIIb but not nisin A. The structure of the 3rd ring of nisin A was quite different from those of the three mutacins, suggesting that the activity of MutHIJ may associate with the specific structure of the 3rd ring from mutacins I, III, and IIIb. Further structural investigation is needed to verify this issue.

In KSM167, we found that *mutFEG* and *mutH* were cotranscribed, but the transcription of the *mutT* gene was independent of that of the *mutFEG* gene (Fig. S4A). In KSM167Δ*mutFEG*, the *P_mutF_* promoter remained, leading to the elevated expression of *mutH* under induction by nisin A (Fig. S4B). In contrast, this increase in the expression of *mutH* could not be seen in KSM167Δ*mutA-G*, in which *P_mutF_* was deleted.

Previously, we demonstrated that the NsrRS TCS regulated the expression of *nsrX* and *mutFEG* by sensing nisin A in the UA159 strain (26), although the *mutE* gene was disrupted to generate two separate genes. In this study, we found that the inducible expression of *mutF* was not observed in the Δ*nsrRS* mutants Nsr-type B, Nsr-type D-I, and Nsr-type E, neither did the inducible expressions of *nsrX* in the Δ*nsrRS* mutants of Nsr-type A and Nsr-type B (Fig. S6). Additionally, the susceptibility to mutacin I in Nsr-type D-I, and to mutacins III and IIIb in Nsr-types B, D-I, and E were elevated when *nsrRS* was deleted (Fig. S4). These results implied that the TCS NsrRS played a role as the basic circuit for activating transcription of *mutFEGHIJ* and *nsrX* under the stimulation of nisin A and mutacins I, III, and IIIb. The similar structures of the first and second rings of these peptides (Fig. 8) may serve as the common inducing signal for the TCS NsrRS.

We also discovered that the rearrangements and modifications of the *mutFEG* cassette within the Nsr region resulted from the insertion of mutacin I, III, or IIIb synthesis genes, along with their modification and transport factors (*mutR ~ mutT*), to confer adaptive resistance against the respective bacteriocins. This modification of *mutFEG* also affected cross-resistance against other bacteriocins, such as nisin A and mutacins. Furthermore, it was interesting to observe that the mutacin I, III, and IIIb peptides utilized the proper TCS and transporters found in all S. mutans strains as immunity factors. Since *mutFEG* and *nsrRS* were present in all isolates irrespective of their mutacin type (in Nsr-type A, *mutFEG* is truncated, although a small portion of *mutF* and *mutG* sequences remain), *mutFEG* and *nsrRS* were considered to have originally been located in S. mutans chromosomal DNA. For the above reasons, we proposed that Nsr-type B showed the original structure, and that Nsr-type A was generated by deletion and/or disruption within the *mutFEG* region (Fig. S7).

Strikingly, *mutFEG* and *nsrRS -* like structures were common in the other mutans group streptococci isolated from humans, chimpanzees, monkeys, bears, and hamsters (Fig. 6), implying a possible transfer of immunity genes among closely related species through double-crossover recombinations. Additionally, the presence of the mobile elements in *S. criceti* and S. mutans Nsr-type E suggests the transmissibility of some immunity factors from one species to another species via transposase activity. The high proportion of isolates showing the resistance against nisin A in our study (>80% with nisin A MIC ≥ 102.4 μg/mL) raises a concern whether a selection of nisin-resistant bacteria has occurred due to the widespread use of nisin as a food preservative. Besides, *S. sobrinus* KCOM1157 was the only species that carries both *mutFEG* and *mutHIJ* (Fig. 6A) and had high sequence identities with MutEG-α (Fig. 6C), while the phylogenetic tree (Fig. 6B) exhibited a relatively distant relationship between S. mutans and *S. sobrinus*, suggesting some meaning of *mutHIJ* exclusively in a portion of S. mutans and *S. sobrinus* population. There is a possibility that this immunity factor originally existed in a far-related species. We found the presence of MutH-like protein in some other species such as *Ligilactobacillus agilis* and *Vagococcus vulneris* (71.95% and 59.17% identity, respectively, Fig. 6C), and MutI-like protein in *Ligilactobacillus agilis* and *Lactiplantibacillus paraplantarum* (49.92% and 32.25% identity, respectively).

Based on our findings, we propose that the chromosomal insertion of bacteriocin genes resulted in the rearrangement and modification of several genes to adapt to the integrated bacteriocins and achieve an optimal balance between the offensive weapons (mutacins) and defensive weapons (immunity factors) of the bacteria themselves. On the other hand, this adaptation sometimes comes at the cost of elevated susceptibility to the other bacteriocins as in the case of Nsr-type D-I. The Nsr-type D-I strains acquired the mutacin I-synthesis operon and produced mutacin I, which effectively kills the other Nsr-types (A, B, d-III, and E). However, to protect themselves against their mutacin, the MutFEG sequence was modified to achieve an optimal function against mutacin I. This modification, in reverse, decreased the specificity with nisin A, mutacin III, and mutacin IIIb, leading to the increased susceptibility against these bacteriocins. Our characterization of immunity factors for nisin A and mutacins in correlation with their amino acid sequences contributes a prediction model for bacteriocin resistance patterns in the other species where homologous sequences could be found. Finally, the evolution and mobility of immunity factors in some bacterial species could give rise to a wide-spectrum resistance in multiple species, which may alter the bacterial flora composition in the host and diminish the effect of bacteriocin as a future therapeutic agent.

## MATERIALS AND METHODS

### Bacterial strains and growth conditions.

S. mutans UA159, S. mutans clinical isolates (18), and Lactococcus lactis ATCC11454 (18) were obtained previously (Table S3). S. mutans and L. lactis strains were grown in Trypticase soy broth (TSB) (Becton, Dickinson and Company, Franklin Lakes, NJ, USA) at 37°C under 5% CO_2_. When necessary, erythromycin (10 μg/mL) or spectinomycin (500 μg/mL) was added to the medium.

### MIC determination.

We used nisin A (Sigma-Aldrich) at a 2.5% concentration. The MIC of nisin A was determined by microdilution in liquid culture (TSB) as described previously (26). S. mutans strains (10^5^ cells) were inoculated into 100 μL of TSB containing various concentrations of nisin A, and MICs were determined after incubation for 24 h at 37°C under 5% CO_2_. To adjust the 2.5% starting concentration, all the experimental MIC values were decreased by 40-fold to obtain the actual nisin A MIC. The actual MIC values were used for all analyses and displayed in the figures.

### Genome sequence analysis.

The genome data of 124 S. mutans strains were obtained previously (18). Based on the gene composition between *alaS* and *nsrS* found in S. mutans UA159, the genes in the Nsr region were identified in 124 S. mutans strains with SnapGene v5.3.2 (https://www.snapgene.com). Comparison of different Nsr structures were performed with BLAST and Easifig v2.2.2 tool. The identities between amino acid sequences were investigated with NCBI BLAST (https://blast.ncbi.nlm.nih.gov/Blast.cgi). The sequences of Nsr region and whole-genome sequences of S. mutans were compared with those from the other streptococcal species including S. mutans UA159 (NC_004350.2), *S. sobrinus* KCOM1157 (NPOU01000001), *S. troglodytae* TKU31 (NZ_AP014612.1), *S. macacae* NCTC11558 (UHFV01000002), S. ratti ATCC31377 (CP043405), *S. ursoris* DSM22768 (NZ_JABASA010000001.1), *S. criceti* NCTC12277 (NZ_UHFB01000008.1), *S. downei* NCTC11391 (NZ_UHFA01000003.1), S. pneumoniae GPSC124 (CAAMIA000000000.1), S. oralis (JAHMJZ010000010.1), *S. parasanuinis* (NZ_GL732449.1), and *S. lactarius* (NZ_CP072329.1).

### Construction of bacterial mutants.

The construction of S. mutans deletion strains and complementation strains was performed with the following protocol. The erythromycin resistance gene (Em^r^) or the spectinomycin gene (Spc^r^) without a terminator was amplified by PCR from pAMβ1 (34) or pDL55 (35), respectively. The deletion mutants were generated by allelic replacement. Approximately 500 bp of the 5′ and 3′-flanking regions of the target gene were amplified by PCR from S. mutans genomic DNA with specific primers. Both PCR amplicons were engineered with complementary sequences for cloning into both ends of the Em^r^ gene or Spc^r^ gene, thus generating a gene cassette comprising an antibiotic resistance gene flanked by the upstream and downstream sequences of the target gene. The resulting PCR amplicon was transformed into S. mutans to produce the corresponding deletion mutant by erythromycin or spectinomycin resistance selection. For genetic complementation, we constructed a gene cassette containing the Em^r^ gene and the full-length coding region of the target gene without its putative promoter region and inserted this fragment into the chromosomal *ftf* gene, which encodes fructosyltransferase. First, the target gene, the ~500 bp fragments upstream and downstream of the *ftf* gene (ftf-UP and ftf-DW, respectively), and the Em^r^ gene were amplified with specific primers containing complementary sequences. Next, overlap extension PCR was carried out, where the complementary sequences facilitated amplicon assembly and ligation. Finally, the resulting PCR amplicon of the whole cassette [fttUP-Em^r^-target gene-ftfDW] was transformed into a deletion mutant (Spc^r^) to generate a complementation strain by erythromycin and spectinomycin resistance selection. The primers used are listed in Table S4.

### Soft agar overlay assay.

To evaluate the antibacterial activity of nisin A, mutacin I, mutacin III, and mutacin IIIb, a soft agar overlay assay was performed. An overnight culture of the bacteriocin-producing strain was spotted onto a TSA plate and cultured at 37°C under 5% CO_2_ for 24 h. After confirming that the diameter of the growth zone of the bacteriocin-producing strain was uniformly 5 mm, 3.5 mL of prewarmed half-strength TSB soft agar (0.75%) containing indicator bacterial cells (10^8^ cells) was poured over the TSA plate. The plates were incubated at 37°C under 5% CO_2_ for 24 h. The diameters of the growth inhibition zones surrounding the bacteriocin-producing strains were measured in three directions. Three independent experiments were performed, and the average diameter was calculated.

### Quantification of gene expression.

Quantitative PCR was performed to investigate the expression of several genes encoding immunity factors and bacteriocin synthesis genes. For the growth of agar medium, a small amount (30 μL) of an overnight culture (10^8^ cells/mL) was spotted on TSA and then grown at 37°C under 5% CO_2_ for 24 h. Bacterial cells were collected in a suspension of sterile PBS (1 mL). For growth in liquid culture, overnight cultures of S. mutans (10^8^ cells) were inoculated into 5 mL of fresh TSB and then grown at 37°C under 5% CO_2_. When the optical density reached 0.5 at 660 nm, nisin A (1/128 MIC) was added to the medium. After 15 min of incubation, bacterial cells were harvested. Total RNA was extracted from the bacterial cells with a FastRNA Pro Blue kit (MP Biomedicals, Solon, OH, USA) according to the manufacturer's protocol, and cDNA was synthesized from 1 μg of total RNA using a first-strand cDNA synthesis kit (Roche, Tokyo, Japan). Subsequently, quantitative PCR was performed with the cDNA as the template DNA using FastStart Essential DNA Green Master and LightCycler 96 Instrument (Roche, Tokyo, Japan). The results were normalized to the housekeeping gene *gyrA* and were analyzed using double delta Ct analysis. The primers used in this study are listed in Table S4. Three independent experiments were performed, and the average value was calculated.

To verify the operons in the region flanked by *alaS* and *nsrS*, we performed PCR using the cDNA of KSM167 induced by nisin A. To amplify the junction between the *mutG* and *mutH* or *mutT* and *mutF* genes, PCR was performed using the specific primers shown in Table S4.

### Phylogenetic trees.

Phylogenetic trees were constructed based on whole-genome sequences using the CSI Phylogeny 1.4 pipeline available from the Center for Genomic Epidemiology (Lungby, Denmark) for SNP calling and was then annotated with Interactive Tree of Life (iTOL) software. The tree was drawn to scale, with branch lengths in the same units as the evolutionary distances used to infer the phylogenetic tree.

### Statistical analysis.

Each experiment was replicated at least 3 times, and the mean value and standard error of the mean (SEM) were calculated. Figures were generated and statistical analysis was performed in GraphPad Prism ver 9.1.0 using one-way ANOVA. For Table 1, Chi-square test and Fisher's exact test were used to generate the *P* value. Significance was defined as *P < *0.05, and *P* value are indicated in graphs and Table 1 as asterisks. * *P < *0.05, ** *P < *0.01, *** *P < *0.001, **** *P < *0.0001.

### Data availability.

The nucleotide sequences of six Nsr types, *mutFEG-α*, *-β*, *and -γ*, and *mutH* and *mutI* have been deposited at NCBI database with accession numbers MZ997347–MZ997352, MZ964799–MZ964801, and MZ964797–MZ964798.
