# Supplementary material for: Gene Rearrangement and Modification of Immunity Factors Are Correlated with the Insertion of Bacteriocin Cassettes in Streptococcus mutans
Source: Microbiol Spectr. 2022 May 23;10(3):e01806-21. doi: 10.1128/spectrum.01806-21 (PMC9241761; doi:10.1128/spectrum.01806-21)
Supplement: SUPPLEMENTAL FILE 1 — Supplemental material. Download spectrum.01806-21-s001.pdf, PDF file, 1.1 MB [file spectrum.01806-21-s001.pdf]

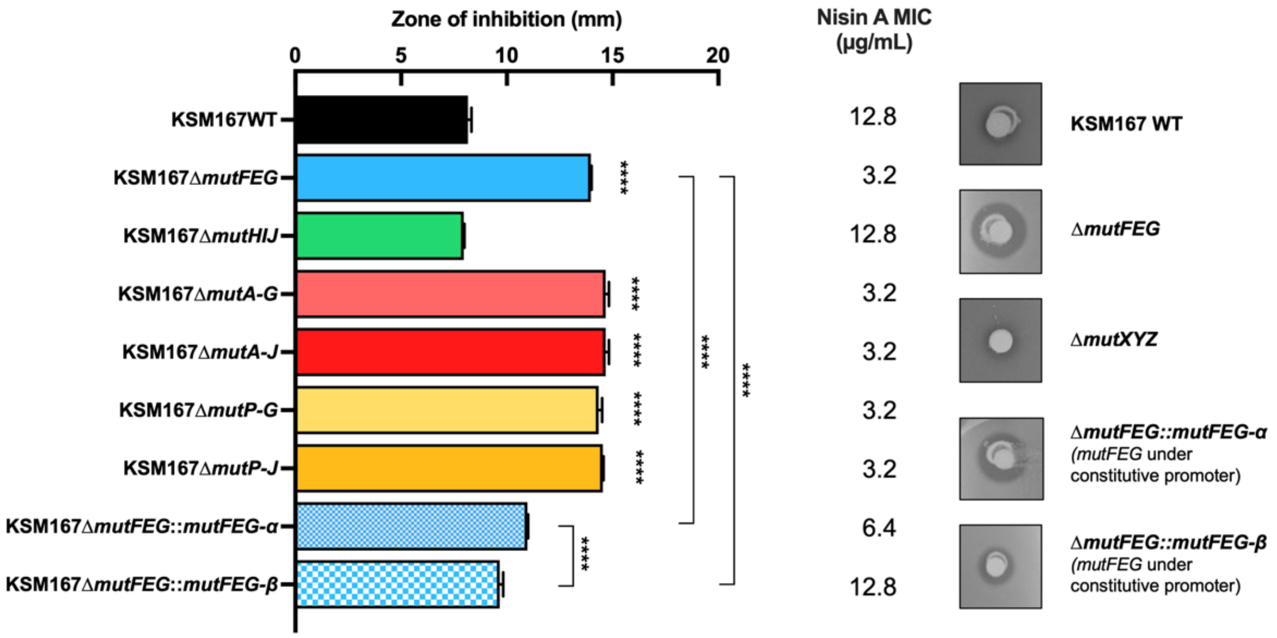

Fig. S1. Immunity factors against nisin A in Nsr-type D-I.

Several KO mutants and complemented strains derived from KSM167 (Nsr-type D-I) were constructed, including Δ*mutFEG*, Δ*mutHIJ,* Δ*mutP-G*, Δ*mutP-J*, Δ*mutA-G*, Δ*mutA-J*, Δ*mutFEG::mutFEG-α*, and Δ*mutFEG::mutFEG-β*. A soft agar overlay assay was performed to evaluate the susceptibility to nisin A in the KSM167 WT and mutant strains. One-way ANOVA was performed to determine statistically significant differences between strains. * $p < 0.05$, ** $p < 0.01$, *** $p < 0.001$, **** $p < 0.0001$.

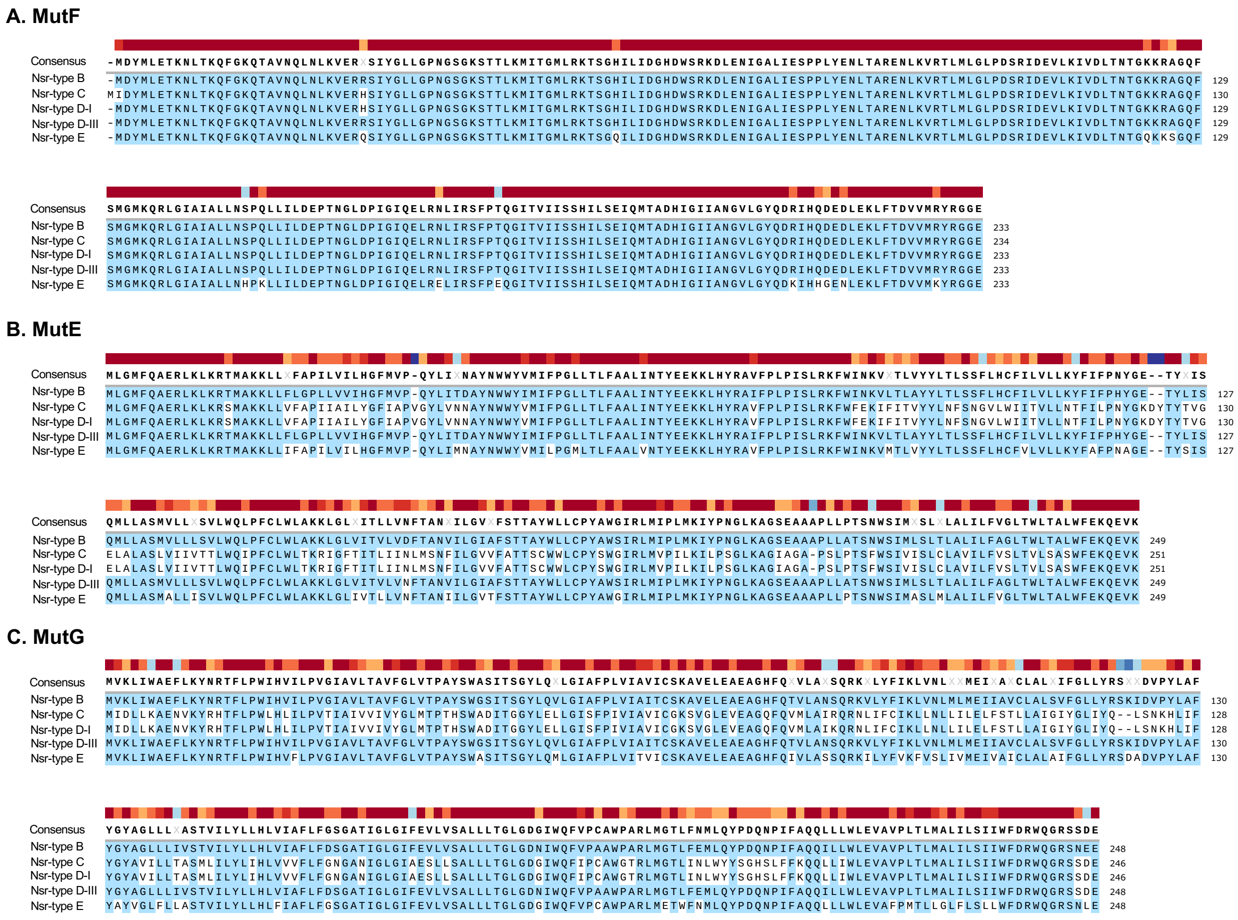

Fig. S2. Comparison of MutFEG sequences among different Nsr types.

(A) Alignment of MutF sequences from Nsr-types B, C, D-I, D-III, and E

(B) Alignment of MutE sequences from Nsr-types B, C, D-I, D-III, and E

(C) Alignment of MutG sequences from Nsr-types B, C, D-I, D-III, and E

A

Nsr types	Mean zone of inhibition (mm)		
	Mutacin I	Mutacin III	Mutacin IIIb
A	15.63	17.26	15.87
B	15.86	13.68	12.59
C	9.720	13.66	12.56
D-I	8.89	13.35	12.31
D-III	12.35	11	12.32
E	15.36	13.06	11.04

B

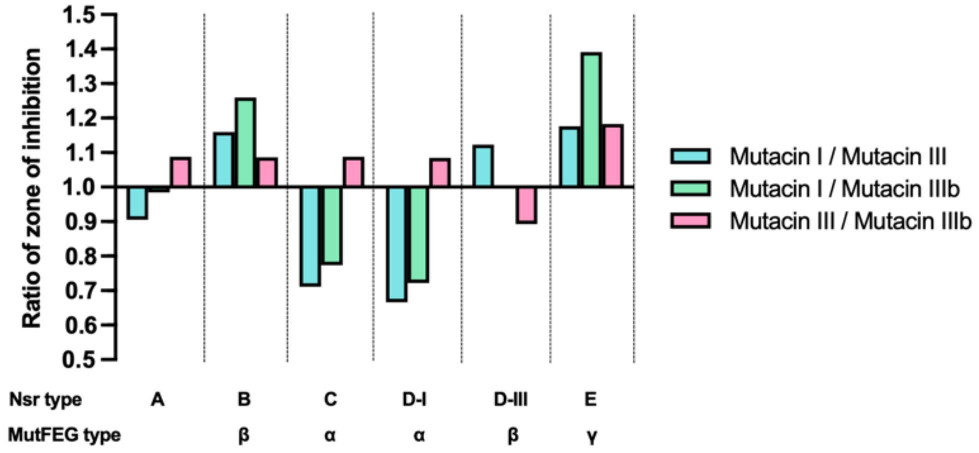

Fig. S3. Comparison of mutacins I, III, and IIIb susceptibility in each Nsr type.

(A) Mean value of the zone of inhibition of each mutacin by Nsr types. The mean values of each Nsr type were calculated from all the strains belonging to that Nsr type.

(B) Pairwise comparison of the mean zone of inhibition of mutacin I, III, and IIIb by Nsr types. The mean values from (A) were used to calculate the ratios of each mutacin pair.

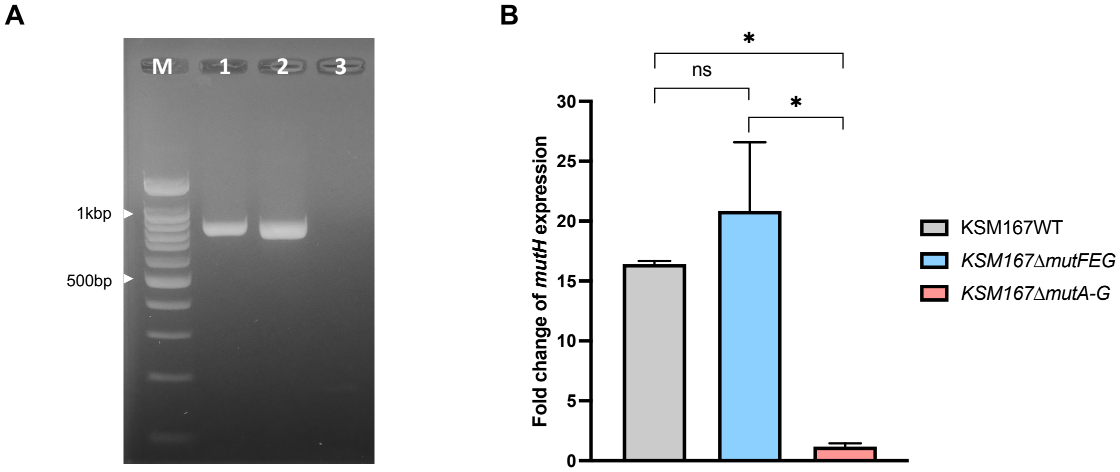

Fig. S4. Transcription of MutHIJ and MutFEG.

(A) MutHIJ and MutFEG are cotranscribed from the same operon. PCR was performed to amplify the junction between *mutG* and *mutH* (lanes 1 and 2) using complementary DNA and chromosomal DNA (as a positive control) of KSM167. The junction between *mutT* and *mutF* was also checked with complementary DNA of KSM167 (lane 3).

(B) Differences in the expression levels of *mutH* in KSM167Δ*mutFEG* and KSM167Δ*mutA~G* when induced with nisin A. * $p < 0.05$.

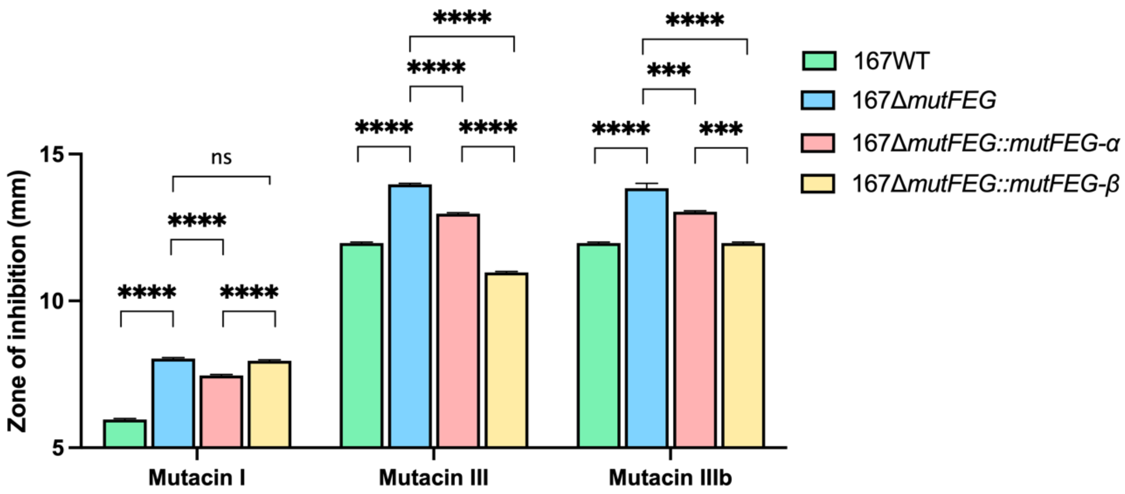

Fig. S5. Mutacin susceptibility among different mutFEG types.

Comparison of mutacins I, III, and IIIb resistance ability between MutFEG-α and MutFEG-β. A soft-overlay assay was performed using the Δ*mutFEG* mutant of KSM167 (Nsr-type D-I) complemented with its own *mutFEG* (*mutFEG-α*) or *mutFEG-β* integrated into the chromosome under the control of a constitutive promoter. KSM167 WT and KSM167Δ*mutFEG* were used as controls. Data are represented as

mean ± SEM (n = 3). One-way ANOVA was performed to determine statistically significant differences between strains. *** $p < 0.001$, **** $p < 0.0001$.

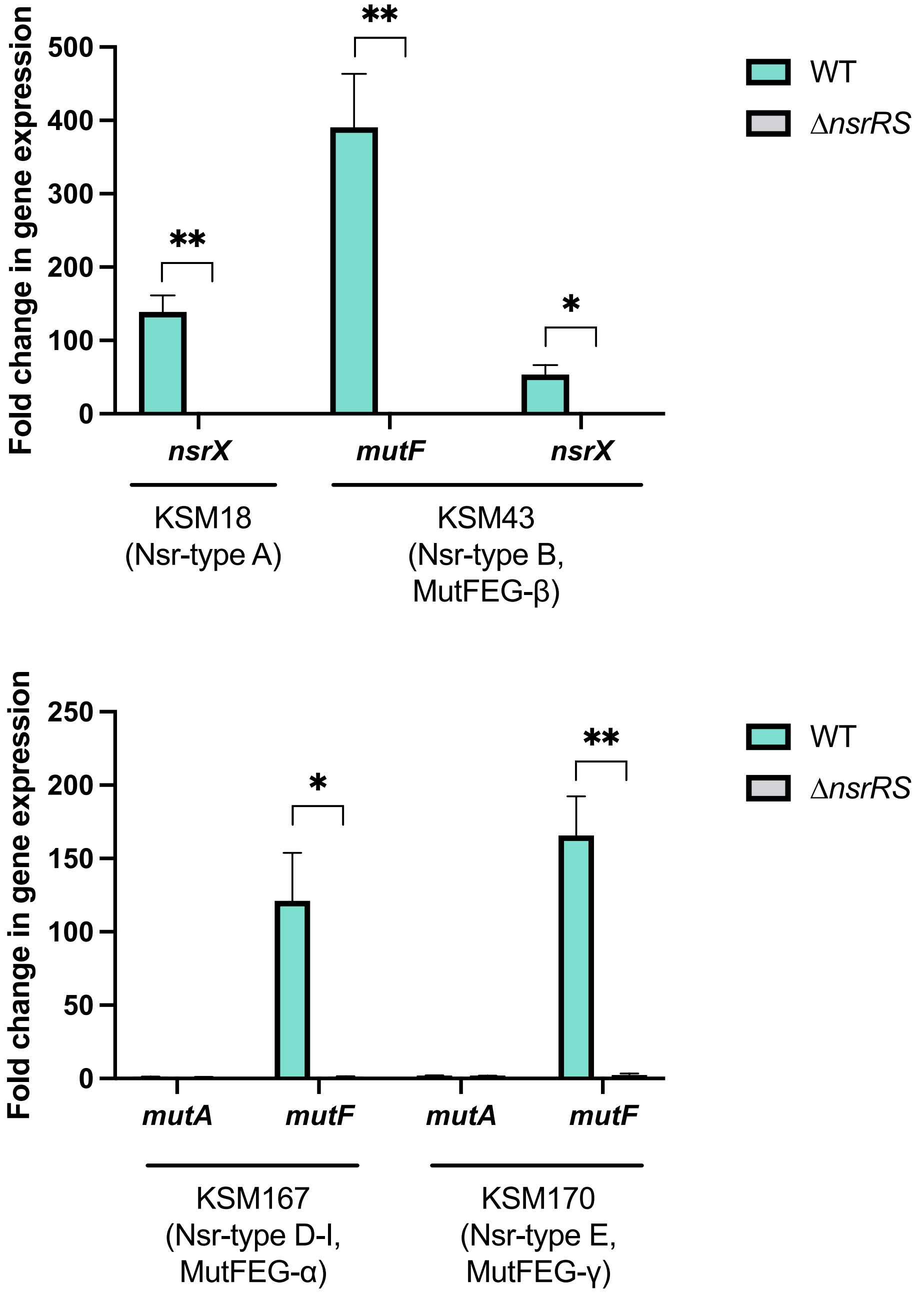

Fig. S6. Expression of *mutF*, *nsrX*, and *mutA* following the addition of nisin A.

(A) Fold change in gene expression of *mutF* and *nsrX* in WT and Δ*nsrRS* of Nsr-types A and B when induced with nisin A.

(B) Fold change in gene expression of *mutF* and *mutA* in WT and Δ*nsrRS* of Nsr-types D-I and E when induced with nisin A.

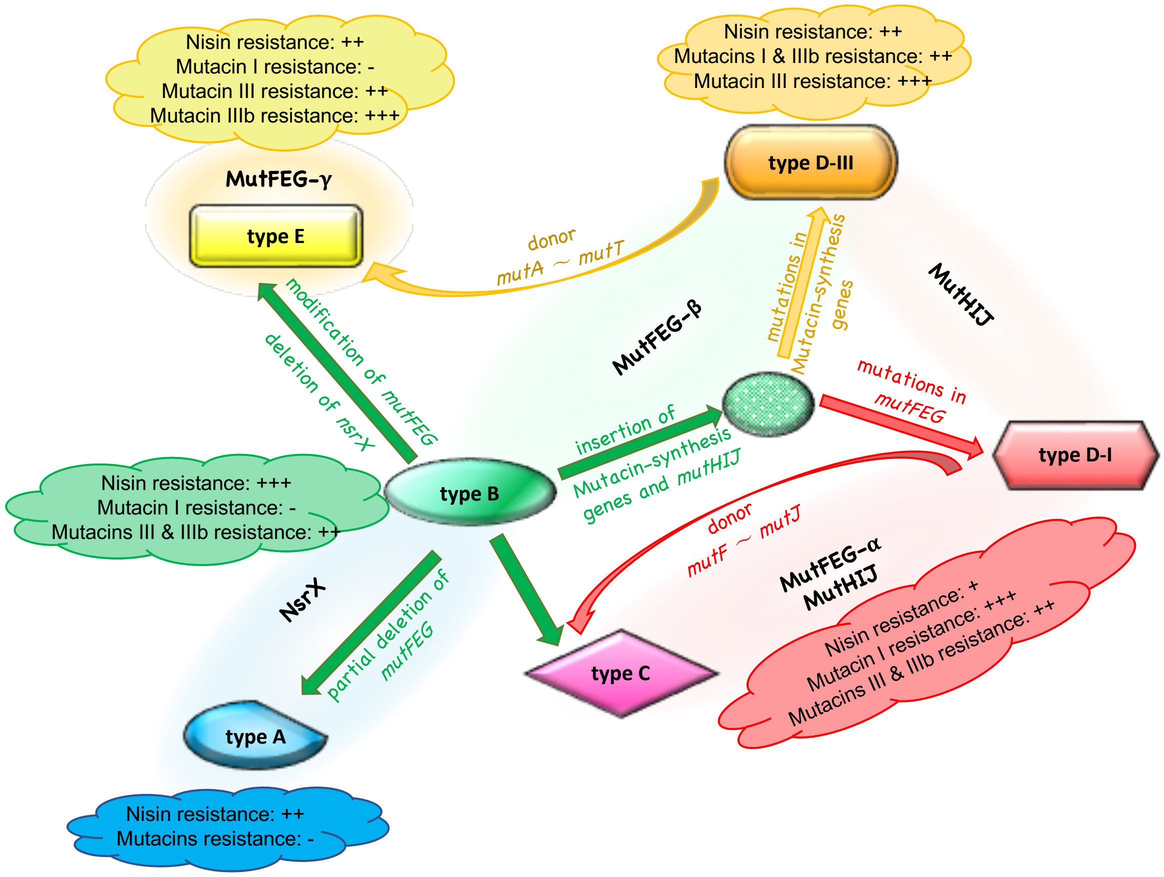

Fig. S7. Hypothetical evolution of the Nsr region.

Nsr-type B is assumed to be the ancestral type among the 6 Nsr types. From Nsr-type B, various Nsr types were generated via the deletion/insertion of some components, resulting in the rearrangement and modification of the Nsr region.

In Nsr-type D-I, the mutacin I synthesis locus and *mutHIJ* cassette might have been independently inserted into an Nsr-type B strain in the regions upstream and downstream of *mutFEG*, respectively, resulting in the reorganization of the Nsr region. Thereafter, the modification of *mutFEG* sequences would have continued until optimal function as an immunity factor against mutacin I was achieved, ultimately generating a new type of MutFEG, designated MutFEG-α. Nsr-type D-III may have been generated in the same manner hypothesized for the evolution of Nsr-type D-I, but instead of modifying the *mutFEG* sequences to adapt to mutacin I, the mutacin synthesis locus (*mutR~mutT)* underwent several mutation events to adapt to the resistance ability of its original immunity factor (MutFEG-β), ultimately giving rise to a new type of mutacin, mutacin III. Nsr-type C possesses *mutFEG* and *mutHIJ* sequences that are identical to those of Nsr-type D-I, suggesting the replacement of *mutFEG* (*mutFEG-β*) and *nsrX* in an Nsr-type B strain with *mutFEG* (*mutFEG-α*) and *mutHIJ* from an Nsr-type D-I strain through double-crossover recombination. Since the mutacin III and IIIb peptides are almost identical, Nsr-type E strains may have been generated by the insertion of the mutacin III-synthesis operon into the Nsr-type B strain. Additionally, two genes encoding IS861 transposases are found upstream of *mutFEG*, inferring that the modification of immunity genes to generate MutFEG-γ in Nsr-type E was facilitated by transposase activity.

Table S1. Correlation between Nsr types and Mutacin types in *S. mutans.*

Nsr type	Mutacin type								Total (%)
	Mutacin I	Mutacin II	Mutacin III	Mutacin IIIb	Mutacin IV	Mutacin Smb	Mutacin K8	No mutacin	
**A**		3			18	15	18	5	49 (39.5%)
**B**					37	11	2	7	46 (37.1%)
**C**					4	3	2	1	5 (4.0%)
**D-I**	17	2							17 (13.7%)
**D-III**			2						2 (1.6%)
**E**				5	3				5 (4.0%)

Table S2. Number of isolates carrying single mutacin, double mutacins, and triple mutacins by Nsr types.

Nsr type	Single mutacin							Double mutacins					Triple mutacins
	Mutacin I	Mutacin II	Mutacin III	Mutacin IIIb	Mutacin IV	Mutacin Smb	Mutacin K8	Mutacin I + II	Mutacin IIIb + IV	Mutacin IV + Smb	Mutacin IV + K8	Mutacin Smb + K8	Mutacin IV + Smb + K8
**A**		3			11	8	14			4	1	1	2
**B**					26	1	1			10	1		
**C**					1					1			2
**D-I**	15							2					
**D-III**			2										
**E**				2					3				

Table S3. Bacterial strains used in this study.

Strains	Description	Source/ Reference
L. lactis ATCC11454	Nisin-producing bacteria	ATCC
S. mutans		
UA159	Genome reference strain	ATCC
KSM strains	Clinical isolates	1
KSM18	*nsrX* - positive *S. mutans*	This study
KSM43	*mutFEG* and *nsrX* - positive *S. mutans*	This study
KSM123	*mutFEG* and *nsrX* - positive *S. mutans*	This study
KSM6	*mutFEG* and *mutHIJ* - positive *S. mutans*	This study
KSM167	Mutacin I - producing, *mutFEG* and *mutHIJ* - positive *S. mutans*	This study
KSM2	Mutacin III - producing, *mutFEG* and *mutHIJ* - positive *S. mutans*	This study
KSM170	Mutacin IIIb - producing, *mutFEG* - positive *S. mutans*	This study
KSM167Δ*mutFEG*	*mutFEG* deletion mutant in KSM167; Ery^r or Spc^r	This study
KSM167Δ*mutHIJ*	*mutHIJ* deletion mutant in KSM167; Ery^r	This study
KSM167Δ*mutP-G*	*mutPTFEG* deletion mutant in KSM167; Ery^r	This study
KSM167Δ*mutP-J*	*mutPTFEGHIJ* deletion mutant in KSM167; Ery^r	This study
KSM167Δ*mutA-G*	*mutABCDPTHIJ* deletion mutant in KSM167; Ery^r	This study
KSM167Δ*mutA-J*	*mutABCDPTFEGHIJ* deletion mutant in KSM167; Ery^r	This study
KSM167Δ*nsrRS*	*nsrRS* deletion mutant in KSM167; Ery^r	This study
KSM170Δ*mutFEG*	*mutFEG* deletion mutant in KSM170; Ery^r	This study
KSM170Δ*mutA-G*	*mutABCDPTFEG* deletion mutant in KSM170; Ery^r	This study
KSM170Δ*nsrRS*	*nsrRS* deletion mutant in KSM170; Ery^r	This study
KSM18Δ*nsrRS*	*nsrRS* deletion mutant in KSM18; Ery^r	This study
KSM43Δ*nsrRS*	*nsrRS* deletion mutant in KSM43; Ery^r	This study
KSM123Δ*mutFEG*	*mutFEG* deletion mutant in KSM123; Ery^r	This study
KSM123Δ*nsrX*	*nsrX* deletion mutant in KSM123; Ery^r	This study
KSM167Δ*mutFEG*::*mutFEG-α*	KSM167Δ*mutFEG* complemented with *mutFEG* derived from KSM167 under constitutive promoter; Spc^r, Ery^r	This study
KSM167Δ*mutFEG*::*mutFEG-β*	KSM167Δ*mutFEG* complemented with *mutFEG* derived from KSM2 under constitutive promoter; Spc^r, Ery^r	This study

Ery^r, erythromycin resistance; Spc^r, spectinomycin resistance

Table S4. Primers used in this study.

Target gene ID	Primer - forward (5' to 3')	Primer - reverse (5' to 3')
**For construction of knockout mutants**		
Δ*mutFEG* in Mutacin I strains	aggttgatgaatatggtgg	cagtcgaggatttcgtctctagcatataatc
	gctgacctagtatttggttcgacagatgg	acctccagataattcataa
Δ*mutHIJ* in Mutacin I strains	aagttattgaatttgctcat	cagtcgaggatagatgactaacaccaatata
	gctgacctagtctttttcgctaataaaaaag	aaatcaagtttctttccctt
Δ*mutP-G* in Mutacin I strains	gaatatattacgtttttgact	cagtcgaggatagttagaaagattattactag
	gctgacctagtatttggttcgacagatgg	acctccagataattcataa
Δ*mutP-J* in Mutacin I strains	gaatatattacgtttttgact	cagtcgaggatagttagaaagattattactag
	gctgacctagtctttttcgctaataaaaaag	aaatcaagtttctttccctt
Δ*mutA-G* in Mutacin I strains	gcttttttgtgattagaag	cagtcgaggatacatcaaaagtttcagtacc
	gctgacctagtatttggttcgacagatgg	acctccagataattcataa
Δ*mutA-J* in Mutacin I strains	gcttttttgtgattagaag	cagtcgaggatacatcaaaagtttcagtacc
	gctgacctagtctttttcgctaataaaaaag	aaatcaagtttctttccctt
Δ*nsrRS* in Mutacin I strains	tattggtttatttgtcattg	cagtcgaggattcatcatcaattaccaaaat
	gctgacctagttgatgctaaaacagccag	caaaagatttagcaatctta
Δ*mutFEG* in Mutacin IIIb strains	ggtctccagatttgaaaca	cagtcgaggattcgtttctagcatatagtc
	gctgacctagtaatatgttatcctagtgaag	tagcagtaacccgcttag
Δ*mutA-G* in Mutacin IIIb strains	gggagaaaaaatgtgtaaaa	cagtcgaggatacatcaaaagtttcagtacc
	gctgacctagtaatatgttatcctagtgaag	tagcagtaacccgcttag
Δ*nsrRS* in Mutacin IIIb strains	ttgctatctgtttagcttta	cagtcgaggattcatcatcaattaccaaaat
	gctgacctagtaatatgttatcctagtgaag	tagcagtaacccgcttag
Δ*nsrRS* in KSM18 and KSM43	attattcttccctttctctt	cagtcgaggattcatcatcaattaccaaaat
	gctgacctagtaatatgttatcctagtgaag	tagcagtaacccgcttag
**For complementation**		
mutFEG from KSM167 or KSM2	gctgacctagtgtccttaaatttgtttttata	cagttagcaggctgagaacgtaatatctttt
**For quantitative PCR**		
mutF in KSM18, 43, and167	ggttttaaaaatagtggatc	ctgaataccaataggatcaa
mutF in KSM170	agtcttaaagattgttgact	ctgaataccaataggatcaa
nsrX in KSM18 and 43	tggaacagcgactttgattg	acctgataaccatacacggc
mutH in KSM167	tgataccagttattatttcg	aatatcaagtctggattcat
mutA in KSM167 (Mutacin I)	tagaagtccttggtactgaa	ttgaaactaggatttttcac
mutA in KSM170 (Mutacin IIIb)	gtccttggtactgaaacttttg	actacctgtctttgcacaacc
gyrA	tctcgctggacttgtcactg	catctaggcgcatcactttg
**For PCR**		
mutG-mutH	cttcaatgctcattctttat	aatatcaagtctggattcat
mutT-mutF	aggttgatgaatatggtgg	ctgaataccaataggatcaa

Underlined sequences denote the complementation sites for gene cloning

Reference

1. Watanabe, A. *et al.* Comprehensive analysis of bacteriocins in *Streptococcus mutans*. *Sci. Rep.* **11**, (2021).
